# Circulating metabolites and the risk of type 2 diabetes: a prospective study of 11,896 young adults from four Finnish cohorts

**DOI:** 10.1007/s00125-019-05001-w

**Published:** 2019-10-04

**Authors:** Ari V. Ahola-Olli, Linda Mustelin, Maria Kalimeri, Johannes Kettunen, Jari Jokelainen, Juha Auvinen, Katri Puukka, Aki S. Havulinna, Terho Lehtimäki, Mika Kähönen, Markus Juonala, Sirkka Keinänen-Kiukaanniemi, Veikko Salomaa, Markus Perola, Marjo-Riitta Järvelin, Mika Ala-Korpela, Olli Raitakari, Peter Würtz

**Affiliations:** 1 Research Centre of Applied and Preventive Cardiovascular Medicine, University of Turku, Kiinamyllynkatu 10, 20520 Turku, Finland; 2 Department of Internal Medicine, Satakunta Central Hospital, Sairaalantie 3, 28500 Pori, Finland; 3 Institute for Molecular Medicine (FIMM), University of Helsinki, Tukholmankatu 8, 00014 Helsinki, Finland; 4 Nightingale Health Ltd, Mannerheimintie 164a, 00300 Helsinki, Finland; 5 Department of Public Health, University of Helsinki, Helsinki, Finland; 6 Centre for Life Course Health Research, Faculty of Medicine, University of Oulu, Oulu, Finland; 7 Computational Medicine, Faculty of Medicine, University of Oulu and Biocenter Oulu, Oulu, Finland; 8 NMR Metabolomics Laboratory, School of Pharmacy, University of Eastern Finland, Kuopio, Finland; 9 Unit of Primary Health Care and Medical Research Center, Oulu University Hospital, Oulu, Finland; 10 Oulunkaari Primary Health Care Unit, Ii, Finland; 11 Medical Research Center Oulu, Oulu University Hospital and University of Oulu, Oulu, Finland; 12 Nordlab Oulu, Oulu University Hospital, Oulu, Finland; 13 Department of Clinical Chemistry, University of Oulu, Oulu, Finland; 14 National Institute for Health and Welfare, Helsinki, Finland; 15 Department of Clinical Chemistry, Fimlab Laboratories, Tampere, Finland; 16 Finnish Cardiovascular Research Center–Tampere, Faculty of Medicine and Health Technology, Tampere University, Tampere, Finland; 17 Department of Clinical Physiology, Tampere University Hospital, Tampere, Finland; 18 Department of Medicine, University of Turku and Division of Medicine, Turku University Hospital, Turku, Finland; 19 Healthcare and Social Services of Selanne, Pyhasalmi, Finland; 20 Diabetes Unit, Healthcare Services of City of Oulu, Oulu, Finland; 21 Estonian Genome Center, University of Tartu, Tartu, Estonia; 22 Department of Epidemiology and Biostatistics, Medical Research Council–Public Health England Centre for Environment and Health, Imperial College London, London, UK; 23 Biocenter Oulu, University of Oulu, Oulu, Finland; 24 Department of Life Sciences, College of Health and Life Sciences, Brunel University, London, UK; 25 Medical Research Council Integrative Epidemiology Unit, University of Bristol, Bristol, UK; 26 Population Health Science, Bristol Medical School, University of Bristol, Bristol, UK; 27 Systems Epidemiology, Baker Heart and Diabetes Institute, Melbourne, Victoria Australia; 28 Department of Epidemiology and Preventive Medicine, School of Public Health and Preventive Medicine, Faculty of Medicine, Nursing and Health Sciences, The Alfred Hospital, Monash University, Melbourne, Victoria Australia; 29 Department of Clinical Physiology and Nuclear Medicine, Turku University Hospital, Turku, Finland; 30 Research Programs Unit, Diabetes and Obesity, University of Helsinki, Helsinki, Finland

**Keywords:** Branched-chain amino acid, Isoleucine, Leucine, Metabolomics, Type 2 diabetes

## Abstract

**Aims/hypothesis:**

Metabolomics technologies have identified numerous blood biomarkers for type 2 diabetes risk in case−control studies of middle-aged and older individuals. We aimed to validate existing and identify novel metabolic biomarkers predictive of future diabetes in large cohorts of young adults.

**Methods:**

NMR metabolomics was used to quantify 229 circulating metabolic measures in 11,896 individuals from four Finnish observational cohorts (baseline age 24–45 years). Associations between baseline metabolites and risk of developing diabetes during 8–15 years of follow-up (392 incident cases) were adjusted for sex, age, BMI and fasting glucose. Prospective metabolite associations were also tested with fasting glucose, 2 h glucose and HOMA-IR at follow-up.

**Results:**

Out of 229 metabolic measures, 113 were associated with incident type 2 diabetes in meta-analysis of the four cohorts (ORs per 1 SD: 0.59–1.50; *p*< 0.0009). Among the strongest biomarkers of diabetes risk were branched-chain and aromatic amino acids (OR 1.31–1.33) and triacylglycerol within VLDL particles (OR 1.33–1.50), as well as linoleic *n*-6 fatty acid (OR 0.75) and non-esterified cholesterol in large HDL particles (OR 0.59). The metabolic biomarkers were more strongly associated with deterioration in post-load glucose and insulin resistance than with future fasting hyperglycaemia. A multi-metabolite score comprised of phenylalanine, non-esterified cholesterol in large HDL and the ratio of cholesteryl ester to total lipid in large VLDL was associated with future diabetes risk (OR 10.1 comparing individuals in upper vs lower fifth of the multi-metabolite score) in one of the cohorts (mean age 31 years).

**Conclusions/interpretation:**

Metabolic biomarkers across multiple molecular pathways are already predictive of the long-term risk of diabetes in young adults. Comprehensive metabolic profiling may help to target preventive interventions for young asymptomatic individuals at increased risk.

**Electronic supplementary material:**

The online version of this article (10.1007/s00125-019-05001-w) contains peer-reviewed but unedited supplementary material, which is available to authorised users.

## Introduction

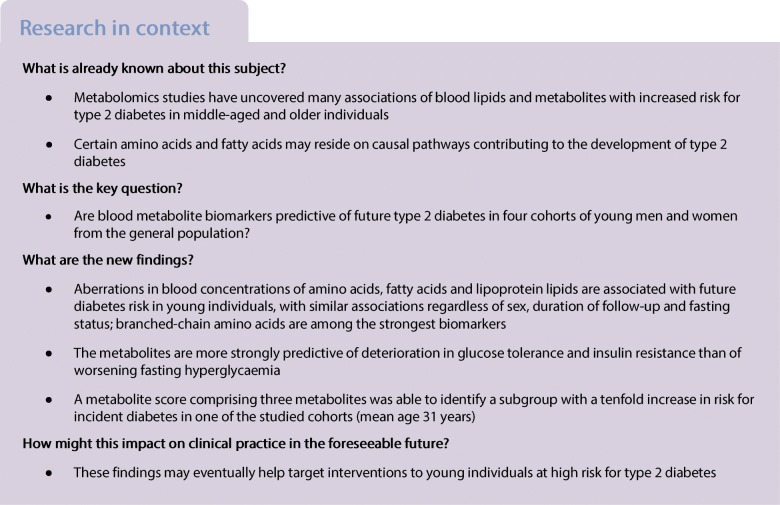



The global prevalence of type 2 diabetes is increasing rapidly, particularly in low- and middle-income countries [1]. Type 2 diabetes is associated with increased mortality risk from vascular and numerous other causes, and reduced quality of life, causing an immense societal cost burden [2, 3]. Given the availability of lifestyle interventions that are effective at preventing or delaying the onset of type 2 diabetes [4, 5], early identification of individuals at high risk is important. The risk for developing type 2 diabetes is, to some extent, reflected in current measures of hyperglycaemia and dyslipidaemia; however, these markers are ineffective for identifying high-risk individuals [6]. This has spurred interest in metabolite profiling technologies, also known as metabolomics, to identify biochemical changes occurring before the onset of diabetes to elucidate the pathophysiology and potentially aid risk prediction for better targeted prevention [7, 8].

Metabolomics is increasingly used in diabetes epidemiology [7, 8]. Multiple case−control studies have identified circulating lipids and metabolites associated with the risk for type 2 diabetes using a range of technological assays, based on MS or NMR [7, 9, 10]. Branched-chain and aromatic amino acids have been observed to be the most consistent metabolite biomarkers for type 2 diabetes [8]. Genetic evidence and experimental studies suggest that impaired metabolism of these amino acids may be causally implicated in the development of insulin resistance and type 2 diabetes [11, 12]. Also, *n*-6 and other fatty acids have emerged as robust biomarkers for future diabetes risk [8, 13, 14]. However, previous metabolomics studies have commonly involved a modest number of participants in nested case−control settings and have almost exclusively been conducted in middle-aged and older individuals.

In this study, we aimed to assess if the metabolic biomarkers are already associated with future onset of type 2 diabetes in young adults, with blood sampling up to 15 years before disease onset. We used NMR metabolomics to quantify 229 metabolic measures in 11,896 individuals from four population-based cohorts with individuals aged 24–45 years at blood draw. The high-throughput NMR platform allows us to validate many known metabolite biomarkers for diabetes and explore novel associations with detailed measures of lipoprotein metabolism. We also assessed of which hyperglycaemia measures the metabolite biomarkers were most strongly reflective, and if a multi-metabolite score would display a stronger association with early risk of type 2 diabetes than any individual metabolite biomarker.

## Methods

### Study populations

**Fig. 1:**
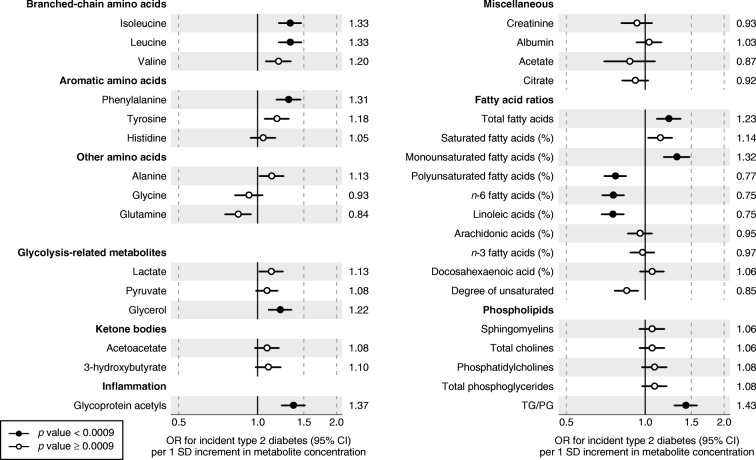
Relationship between baseline circulating metabolite concentrations and risk of future type 2 diabetes. Values are ORs (95% CIs) per 1 SD log_*e*_-transformed metabolite concentration. ORs were adjusted for sex, baseline age, BMI and fasting glucose. The results were meta-analysed for 11,896 young adults from four prospective cohorts. PG, phosphoglyceride; TG, triacylglycerol

The study involved 11,896 individuals from four prospective population-based cohorts in Finland. An overview of the study cohorts and participants included in the present analyses is shown in electronic supplementary material (ESM) Fig. 1. Details of the individual cohorts are provided in ESM Methods. All participants gave written informed consent and the studies were approved by local ethics committees. In all cohorts, we excluded individuals with diabetes at baseline, pregnant women, study participants aged over 45 years at the blood draw and those lacking follow-up information on diabetes diagnosis. The characteristics of each cohort are described in brief below.

#### Cardiovascular Risk in Young Finns Study

In the Cardiovascular Risk in Young Finns Study (YFS), serum metabolites were quantified from 2248 individuals in the 2001 survey. The final sample consisted of 2141 individuals in the age range 24–39 years. The follow-up time was 10 years. Type 2 diabetes diagnoses at 10 year follow-up were based either on HbA_1c_ or fasting glucose assessed in the 2011 re-survey or nationwide registers of reimbursement for diabetes medication or inpatient hospital ICD-10 diagnosis of diabetes (http://apps.who.int/classifications/icd10/browse/2016/en; see ESM Methods) [15].

#### FINRISK-1997

Serum metabolites were quantified from 7603 individuals. The final sample consisted of 3063 individuals when limiting analyses to participants aged 24–45 years. The follow-up time was 15 years. Type 2 diabetes diagnoses at follow-up were based on nationwide register data [16].

#### Dietary, Lifestyle, and Genetic Determinants of Obesity and Metabolic Syndrome Study

In the Dietary, Lifestyle, and Genetic Determinants of Obesity and Metabolic Syndrome Study (DILGOM), serum metabolites were quantified from 4816 individuals in 2007. The final sample consisted of 1421 individuals when limiting analyses to participants in the age range 25–45 years. The follow-up time was 7.8 years. Type 2 diabetes diagnoses at follow-up were based on fasting glucose at the re-survey conducted in 2014 or nationwide register data.

#### Northern Finland Birth Cohort

In the Northern Finland Birth Cohort (NFBC) of 1966, serum metabolites were quantified from 5680 individuals in the 1997 survey. The final sample consisted of 5275 individuals aged 30–32 years. The follow-up time was 15 years. Type 2 diabetes diagnoses were based on either fasting or 2 h glucose at the 46 year follow-up conducted in 2012 or nationwide register data.

### Metabolite quantification

A high-throughput NMR metabolomics platform (Nightingale Health, Helsinki, Finland) was used to quantify 229 metabolic measures from baseline serum samples [17]. This metabolite panel captures a range of established and emerging biomarkers from multiple metabolic pathways, including amino acids, glycolysis-related metabolites, fatty acids and detailed lipoprotein lipid profiles, covering triacylglycerol, total cholesterol, non-esterified cholesterol, esterified cholesterol and phospholipids within 14 subclasses. The same experimental NMR setup and software library was used for metabolite quantification for all four cohorts. The mean levels and distributions of metabolite concentrations were coherent across the cohorts [18]. Details of the NMR metabolomics experimentation have been described previously [17] and epidemiological applications have recently been reviewed [7].

### Statistical analyses

Owing to the skewness of the metabolite distributions, all metabolite concentrations were log_*e*_(metabolite+1) transformed prior to analyses and scaled to SD concentrations separately for each cohort. Although 229 metabolic measures in total were analysed, the number of independent tests performed is lower because of the correlated nature of the measures [7]. We calculated that 54 principal components explained 99% of the variation in the metabolic measures. Alternative methods have yielded a similar number of independent tests in the NMR metabolite data [19, 20]. Hence, we inferred statistical significance at meta-analysis *p* value <0.0009 (0.05/54). The ORs of 229 circulating metabolic measures with incidence of type 2 diabetes were assessed using logistic regression. Each metabolite was analysed for association with incident diabetes in a separate model, adjusted for sex, baseline age, fasting glucose and BMI. To facilitate comparison of the magnitudes of biomarker association for measures with units and different concentration ranges, the ORs are scaled to 1 SD increments in log_*e*_-transformed metabolite concentration. Results from individual cohorts were combined using inverse variance-weighted fixed-effect meta-analysis. We also assessed the influence of additional adjustment for HOMA-IR index, tested results separately for men and women and compared the pattern of metabolite associations with incident type 2 diabetes with that of impaired fasting glucose (≥6.0 mmol/l) at follow-up.

Metabolite associations were also assessed cross-sectionally with BMI, HOMA-IR and fasting glucose using linear regression models adjusted for age and sex, and prospectively with fasting glucose, 2 h glucose, HbA_1c_ and HOMA-IR at follow-up, adjusting for sex, baseline age, fasting glucose and BMI.

Last, we examined the association with future diabetes risk using a multi-metabolite score, composed as the weighted sum of metabolite concentrations. The metabolite selection and weights in the multi-metabolite score were derived by meta-analysis of three of the cohorts (YFS, FINRISK-1997 and DILGOM, constituting approximately half of the incident cases) using forward stepwise logistic model testing of all metabolites. Age, sex, baseline fasting glucose and BMI were always included as covariates in the models for metabolite selection. In each step, the metabolite with the lowest *p* value was added as a covariate, and associations of all remaining metabolites with diabetes risk were assessed. This process was repeated until no further metabolites were significant at *p*< 0.0009 in meta-analysis of the three derivation cohorts. The multi-metabolite score was defined as the sum of concentrations of the three selected metabolites weighted by β-coefficients in the final stepwise model. This multi-metabolite score was then evaluated for association with diabetes risk in NFBC, as this cohort had the highest number of cases and most reliably ascertained diagnoses. ORs of the multi-metabolite score were assessed both as a continuous marker and by quintile, with adjustment for sex, baseline age, fasting glucose and BMI. The influence of further adjustment for HOMA-IR, triacylglycerol and HDL-cholesterol was also assessed. The risk discrimination when adding the multi-metabolite score to models containing these two sets of clinical variables were compared in terms of C-statistic, integrated discrimination improvement and continuous reclassification [21]. Statistical analyses were performed in R version 3.1.3 (R Foundation for Statistical Computing, Vienna, Austria; https://www.R-project.org/).

## Results

**Fig. 2:**
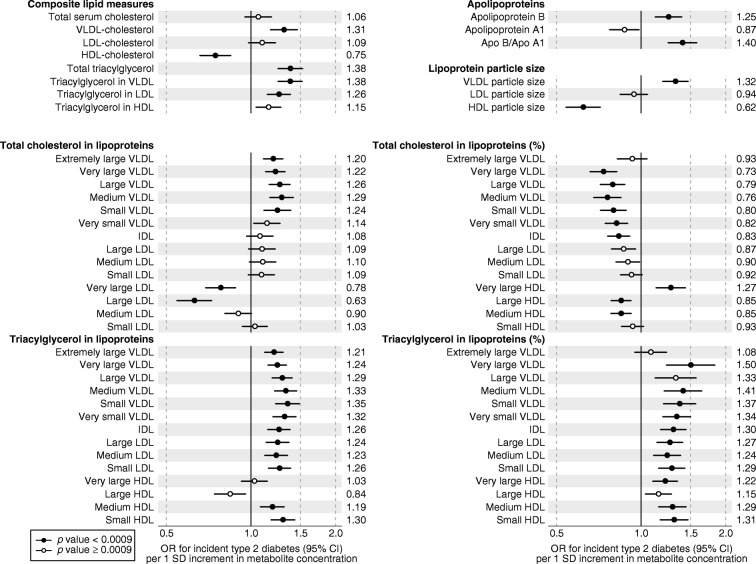
Relationship between baseline circulating lipoprotein measures and risk of future type 2 diabetes. Values are ORs (95% CIs) per 1 SD log_*e*_-transformed metabolite concentration. ORs were adjusted for sex, baseline age, BMI and fasting glucose. The results were meta-analysed for 11,896 young adults from four prospective cohorts. ORs for the remaining 125 metabolic measures assayed are shown in ESM Fig. 2. ApoA1, apolipoprotein A1; ApoB, apolipoprotein B

The study included 11,896 individuals from four Finnish cohorts. The characteristics of the study participants at the time of blood sampling are shown in Table 1. The mean age was 32.9 years (range 24–45 years). The follow-up time ranged from 8 to 15 years, during which a total of 392 incident cases of type 2 diabetes occurred. Mean concentrations and SDs of all metabolic measures are listed in ESM Table 1. The ORs of 104 selected metabolic measures with incident type 2 diabetes are shown in Figs 1 and 2; results for the remaining 125 metabolic measures assayed are found in ESM Fig. 2. In meta-analysis of all four cohorts, 113 out of the 229 metabolic measures were robustly associated with incident type 2 diabetes (*p*< 0.0009) when adjusting for sex, baseline age, BMI and fasting glucose. The biomarkers associated with risk of future type 2 diabetes risk spanned multiple metabolic pathways of polar metabolites, fatty acids and detailed lipoprotein lipid measures, with significant ORs ranging from 1.18 to 1.50 for direct associations and from 0.59 to 0.86 for inverse associations per 1 SD metabolite concentration.

**Table 1:** Baseline characteristics of participants in the four prospective cohorts

Characteristic	YFS	FINRISK-1997	DILGOM	NFBC
Number of individuals	2141	3063	1421	5271
Number of incident type 2 diabetes cases	65	110	18	199
Follow-up time (years)	10	15	8	15
Sex (% women)	53.8	52.7	55.7	50.2
Baseline age (years)	31.7 ± 4.7	35.3 ± 6.0	35.7 ± 6.2	31.2 ± 0.4
BMI (kg/m^2^)	25.0 ± 4.4	25.1 ± 4.2	25.6 ± 4.4	24.6 ± 4.1
Glucose (mmol/l)	5.0 ± 0.4	4.7 ± 0.6	5.6 ± 0.4	5.0 ± 0.4
Total cholesterol (mmol/l)	5.1 ± 1.0	5.1 ± 1.0	5.0 ± 0.9	5.0 ± 1.0
HDL-cholesterol (mmol/l)	1.3 ± 0.3	1.4 ± 0.3	1.4 ± 0.4	1.5 ± 0.4
Triacylglycerol (mmol/l)	1.3 ± 0.8	1.3 ± 1.0	1.3 ± 0.9	1.2 ± 0.7
Plasma insulin (pmol/l)	52.8 ± 36.1	39.6 ± 38.2	38.9 ± 27.1	57.6 ± 27.1
Lipid-lowering medication (%)	0.3	0.3	1.3	0.1

Values are mean ± SD

### Amino acids, glycolysis and inflammation

The branched-chain amino acids isoleucine, leucine and valine (ORs 1.20–1.33) and the aromatic amino acids phenylalanine and tyrosine (ORs 1.31 [95% CI 1.18, 1.46] and 1.18 [95% CI 1.06, 1.32], respectively) were associated with the risk of type 2 diabetes (Fig. 1). Glycerol was also associated with increased risk (OR 1.22 [95% CI 1.10, 1.35]), while other glycolysis-related metabolites had weaker associations. The inflammatory biomarker glycoprotein acetyls (GlycA) displayed one of the strongest associations for type 2 diabetes risk (OR 1.37 [95% CI 1.24, 1.51]).

### Fatty acids

The total concentration of circulating fatty acids (OR 1.23 [95% CI 1.11, 1.36]) and the relative amount of monounsaturated fatty acids ([MUFA] ratio to total fatty acids) were directly associated with increased risk for type 2 diabetes (OR 1.32 [95% CI 1.18–1.48]). In contrast, higher relative concentrations of *n*-6 fatty acids were associated with decreased risk for type 2 diabetes (OR 0.75 [95% CI 0.69, 0.83]). This inverse association was primarily driven by linoleic acid, whereas the association for arachidonic acid was weaker.

### Lipoprotein measures

Both lipid measures used in routine clinical settings and more fine-grained lipoprotein subclass measures were quantified by the NMR metabolomics platform. The associations of routine lipids, as well as cholesterol and triacylglycerol concentrations in 14 lipoprotein subclasses, with type 2 diabetes risk are shown in Fig. 2. Additional lipoprotein subclass measures are shown in ESM Fig. 2.

Overall, the cholesterol concentration within VLDL particles was associated with increased risk for type 2 diabetes, whereas the cholesterol in HDL particles was associated with decreased risk. Cholesterol in very large and large HDL particles was particularly strongly associated with decreased diabetes risk. The association patterns were similar for non-esterified cholesterol and cholesteryl esters; the strongest biomarker for decreased diabetes risk was non-esterified cholesterol in large HDL (OR 0.59 [95% CI 0.50, 0.68]; ESM Fig. 2. However, this pattern of lipoprotein lipid association was different for triacylglycerols: increased triacylglycerol concentrations in all VLDL, intermediate-density lipoprotein (IDL) and LDL as well as medium-sized and small HDL subclasses were strongly associated with increased type 2 diabetes risk. The prominent importance of triacylglycerols was also evident when examining the associations for the relative fraction of triacylglycerol in each lipoprotein subclass, i.e. the percentage of triacylglycerol per total lipid concentration in a given size of lipoprotein particle: a higher relative abundance of triacylglycerols within lipoprotein particles was strongly associated with higher diabetes risk (Fig. 2). Because a higher relative triacylglycerol content in lipoprotein particles generally reflects a lower cholesterol content, then the relative fraction of cholesterol in most lipoprotein subclasses was inversely associated with future diabetes risk.

Concentration of apolipoproteins, the structural proteins of lipoprotein particles, was also associated with increased risk for type 2 diabetes. In particular, the ratio of apolipoprotein B to apolipoprotein A1 was among the strongest predictors (OR 1.40 [95% CI 1.25, 1.58]). Further, larger VLDL particle size was associated with increased diabetes risk (OR 1.32 [95% CI 1.19, 1.47]), whereas larger HDL particle size displayed an inverse association (OR 0.62 [95% CI 0.54, 0.72]).

### Consistency across cohorts and influence of adjustment for insulin resistance

The patterns of association between metabolites and incident type 2 diabetes were highly consistent in all four cohorts despite between-cohort differences in fasting status and ascertainment of diabetes diagnoses at follow-up (ESM Fig. 3). The metabolite associations were highly similar for men and women (ESM Fig. 4). Most associations between metabolites and future risk of type 2 diabetes were moderately attenuated when including HOMA-IR as covariate, but the overall pattern persisted and 71 of the metabolic measures remained significant at *p*< 0.0009 (ESM Fig. 5). Results were almost identical if random-effects rather than fixed-effects were used in meta-analyses and if time-to-event Cox models were used instead of logistic regression (ESM Table 2).

### Prospective metabolite associations with measures of hyperglycaemia

**Fig. 3:**
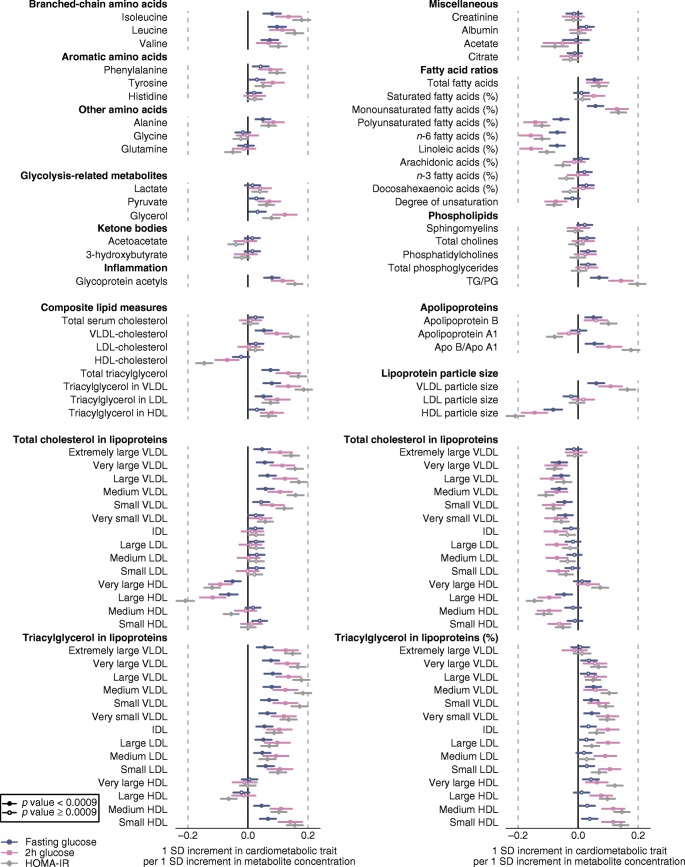
Relationship between baseline circulating metabolites and lipids to blood glucose measures at follow-up. The prospective associations were assessed for fasting glucose (*n* = 5017), 2 h glucose (*n* = 3028) and HOMA-IR (*n* = 5010). Values are β-coefficients (95% CIs) scaled to 1 SD in each of the measures of blood glucose per 1 SD log_*e*_-transformed metabolite concentration. Associations were adjusted for sex, baseline age, BMI and fasting glucose. ApoA1, apolipoprotein A1; ApoB, apolipoprotein B; PG, phosphoglyceride; TG, triacylglycerol

To clarify the aspects of hyperglycaemia reflected most closely by the observed metabolic aberrations, we examined the metabolite associations with fasting glucose, 2 h glucose and HOMA-IR measured in the follow-up surveys 8–15 years after the baseline (Fig. 3). The overall pattern of metabolite associations was similar for the three continuous measures of blood glucose; however, the magnitudes of associations were, on average, 2.2-fold stronger for HOMA-IR and 1.7-fold stronger for 2 h glucose compared with association magnitudes for fasting glucose (ESM Fig. 6). Consistently, the ORs were almost twice as strong for metabolite associations with incident type 2 diabetes compared with incident impaired fasting glucose (≥6.0 mmol/l at follow-up; ESM Fig. 7). In line with these prospective analyses, we found that the metabolite associations were strongly associated with HOMA-IR and BMI as assessed cross-sectionally, whereas the associations with fasting glucose at baseline were substantially weaker in these young adults (ESM Fig. 8).

### Multi-metabolite score strongly associates with future diabetes

To examine if a combination of metabolites would be more strongly associated with diabetes risk than any individual metabolite biomarker, we derived a multi-metabolite score. The weights for adding up the metabolite concentrations in the multi-metabolite score were derived using a stepwise modelling approach based on three of the cohorts. In this manner, three metabolic measures were selected as independent predictors of diabetes: phenylalanine, non-esterified cholesterol in large HDL and cholesteryl ester to total lipid ratio within large VLDL. The association of this multi-metabolite score was then evaluated separately in the NFBC study: the multi-metabolite score was more strongly associated with incident type 2 diabetes than any individual metabolite measure (OR 1.76 [95% CI 1.48, 2.09] per SD]). When dividing individuals based on quintiles of their multi-metabolite score, the OR was 10.1 (95% CI 4.2, 24.1) among individuals in the upper fifth compared with those in the lower fifth when adjusting for age, sex and baseline glucose and BMI (Table 2). If further adjusting for baseline HOMA-IR, triacylglycerol and HDL-cholesterol, the OR for the highest vs lowest fifth of the multi-metabolite score was attenuated to 5.79 (95% CI 2.22, 15.1). The discrimination in absolute risk for future type 2 diabetes is presented in ESM Tables 3–5 and ESM Fig. 9.

**Table 2:** Multi-metabolite score for the risk of type 2 diabetes during the 15 year follow-up, assessed for 5271 individuals aged 31 years at blood sampling

	Incident type 2 diabetes cases^a^, *n* (%)	OR (95% CI)	
		Model 1^b^	Model 2^c^
Score quintile			
Lower fifth	6 (0.6)	Reference	Reference
20–40%	14 (1.3)	2.17 (0.83, 5.67)	1.95 (0.74, 5.16)
40–60%	36 (3.4)	4.09 (2.08, 12.0)	3.93 (1.59, 9.70)
60–80%	47 (4.5)	5.92 (2.47, 14.2)	4.11 (1.63, 10.3)
Upper fifth	96 (9.1)	10.1 (4.20, 24.1)	5.80 (2.22, 15.1)
Per 1 SD increment		1.76 (1.48, 2.09)^d^	1.42 (1.14, 1.76)^e^

The multi-metabolite score was calculated as the weighted sum of concentrations of three circulating metabolites: phenylalanine (weight 0.320), non-esterified cholesterol in large HDL (weight −0.474) and ratio of cholesteryl esters to total lipids in large VLDL (weight −0.321). The β-coefficients used as weights for the biomarkers score were derived by meta-analysis of three derivation cohorts

^a^The lower fifth quantile contains 1055 individuals and the other quantiles 1054 individuals

^b^With age, sex, BMI and fasting glucose as covariates

^c^Model 1 + triacylglycerol, HDL-cholesterol and HOMA-IR as additional covariates

^d^*p* = 2× 10^−10^

^e^*p* = 0.002

## Discussion

This large multi-cohort study describes the metabolic signature of increased type 2 diabetes risk in young adults up to 15 years prior to disease onset. Metabolic aberrations related to incident type 2 diabetes spanned amino acids, fatty acid balance, inflammation and detailed lipoprotein particle composition, with consistent results across the four cohorts. Many of these metabolic measures have previously been associated with future diabetes in middle-aged and older individuals. Among the strongest biomarkers were higher concentrations of branched-chained and aromatic amino acids, VLDL particle measures and the enrichment of triacylglycerol in all lipoprotein subclasses. Moreover, higher circulating levels of GlycA, glycerol and MUFA were also associated with increased risk for type 2 diabetes, whereas glutamine, linoleic acid, HDL particle size and certain lipid measures within large HDL were associated with lower risk. These metabolic aberrations were more strongly predictive of deterioration of insulin sensitivity and impaired post-load glucose levels over long-term follow-up than worsening of fasting hyperglycaemia. A multi-metabolite score consisting of three metabolic measures was associated with a tenfold elevation in the long-term risk for type 2 diabetes in one of the cohorts, comprising 31-year-old men and women.

The metabolic signature for type 2 diabetes risk described here included biomarkers across multiple molecular pathways. Branched-chain and aromatic amino acids were among the first biomarkers for type 2 diabetes risk identified by metabolomics [10]. Their association with future diabetes has since been replicated in several epidemiological studies [8, 9, 22] and extended to insulin resistance and blood glucose [9, 23, 24]. The ORs of all amino acids assayed in this study were consistent with a recent meta-analysis of prospective studies [8]. We extend these prior findings by showing that branched-chain and aromatic amino acid levels already associate with the long-term risk of type 2 diabetes in young adults. Our results also show that the perturbed amino acid levels are more strongly indicative of future impaired glucose tolerance and insulin resistance than of worsening in fasting glucose levels.

The mechanistic underpinnings and causal relation between amino acid levels and type 2 diabetes risk are not yet fully clear [25]. Mendelian randomisation studies have indicated that adiposity and insulin resistance lead to increased branched-chain amino acid levels [12, 26]; other Mendelian randomisation studies suggest that the metabolism of these amino acids may play a causal role in the development of type 2 diabetes [11]. In addition, physiological studies have suggested mechanisms by which alterations in branched-chain amino acid metabolism might cause insulin resistance and impairment of insulin secretion [27, 28]. Altered amino acid metabolism may also represent a link between diabetes and cardiovascular diseases [29, 30]. Our results in young adults support the notion that amino acid profiling may prove helpful for monitoring cardiometabolic health in asymptomatic individuals, with the potential to facilitate targeted interventions [31].

Increasing evidence suggests that levels of certain fatty acids are associated with type 2 diabetes risk. Our finding that a higher relative concentration of *n*-6 fatty acids was associated with decreased diabetes risk, whereas higher MUFA levels were associated with increased diabetes risk is consistent with previous investigations [13, 14]. Consistent with our results in young adults, a recent study from 20 prospective cohorts reported that higher levels of linoleic acid in serum and different lipid compartments is associated with lower risk of type 2 diabetes [14]. The circulating fatty acid biomarkers are reflective of both dietary composition and endogenous metabolism [32]. Dietary counselling aiming to replace saturated fat with unsaturated fat in the diet, in accordance with Nordic dietary recommendations, has been shown to decrease circulating MUFA and increase circulating *n*-3 and *n*-6 levels [33]. If these fatty acids play a causal role in the development of type 2 diabetes, then our results suggest that interventions modifying the circulating fatty acid composition could be effective in prevention.

Pervasive alterations in the lipoprotein profile were also found to be associated with future diabetes risk. These included both established lipids and novel findings based on detailed lipoprotein subclass measures. The lipid modulations shown here to reflect diabetes risk in young adults are similar to those previously reported in cross-sectional settings for older individuals with impaired glucose tolerance [34, 35]. The results for VLDL and HDL particle size are consistent with a large study of American women [36]. In addition, we report novel associations of lipoprotein composition, showing increased risk associated with a higher relative fraction of triacylglycerol in VLDL, LDL as well as HDL. Higher percentage triacylglycerol in VLDL subclasses was associated with the strongest increase in diabetes risk among all metabolic measures assayed. These results reflect early stages of the aberrations in lipoprotein metabolism characteristic of insulin resistance: increased production of large VLDLs, increased catabolism of HDLs and increased transfer of triacylglycerol to HDL and LDL particles [37]. Consistent with this, we showed that the lipoprotein lipid perturbations were strongly reflective of future insulin resistance and impaired glucose tolerance. Our findings indicate that such distortions of lipoprotein metabolism may already be present in normoglycaemic young adults and reflect an increased risk for insulin resistance and type 2 diabetes.

In addition to modulations in lipoprotein metabolism, metabolic measures related to lipolysis (glycerol) and inflammation (GlycA, a marker of chronic inflammation [38, 39]) were predictive biomarkers, illustrating that many different pathways are perturbed long before the onset of type 2 diabetes. The overall metabolic signature of increased diabetes risk was reminiscent of the patterns of metabolite associations for adiposity and insulin resistance index, cross-sectionally and prospectively. This is keeping with previous large-scale metabolic profiling studies [23, 24, 26] and consistent with the pathophysiology of type 2 diabetes, where insulin sensitivity gradually declines years before clinical disease onset [40]. It suggests that the metabolic biomarkers for type 2 diabetes are predominantly manifestations of developing insulin resistance. Nonetheless, the overall pattern of biomarker associations remained predictive after controlling for baseline BMI and HOMA-IR. These results indicate that metabolomic profiling is sensitive to subtle metabolic changes that precede insulin resistance and hyperglycaemia in apparently healthy young adults.

Whereas the comprehensive signature of single biomarkers for type 2 diabetes risk provides a picture of the numerous metabolic pathways reflective of the disease development, the measurement of multiple biomarkers in one go may prove beneficial for disease prediction. We found that a simple multi-metabolite score comprised of phenylalanine and two detailed lipoprotein measures was a stronger predictor of diabetes risk than any of the individual biomarkers. The tenfold elevation in diabetes risk observed here for those in the highest fifth compared with the lowest fifth of the multi-metabolite score indicates that multi-metabolite scores hold potential to aid identification of high-risk individuals at a young age. Future studies with a larger number of incident diabetes cases are needed to evaluate the potential of such scores for risk identification and health tracking in clinical settings.

Our study has both strengths and limitations. Its strengths include the large sample size and the profiling of multiple prospective cohorts. Our results were consistent across cohorts despite differences in age distribution, fasting status and diagnostic ascertainment. The study design allowed derivation and validation of the multi-metabolite score in independent cohorts. Some limitations also need to be considered. First, because type 2 diabetes is relatively rare among young adults, the number of cases was modest despite the large sample size. The power for evaluating the predictive value of the multi-metabolite score was therefore limited. Second, as all cohorts were Finnish, our results cannot necessarily be generalised beyond white Europeans. However, previous research shows that amino acid measures may be even stronger predictors of type 2 diabetes in South Asians compared with Europeans [41]. Third, the NMR metabolomics platform is not able to quantify metabolites present in blood in very low concentrations, and therefore we could not replicate several previously reported metabolomic biomarkers for diabetes [8, 9, 42]. Nonetheless, the NMR metabolomics method is high-throughput and consistent over time, and therefore it is particularly suited for large cohorts. We acknowledge the lack of coherent dietary information across the cohorts and that a large fraction of the samples were non-fasting; however, we observed highly consistent biomarker associations between cohorts with fasting samples and the FINRISK 1997 cohort with non-fasting samples.

In conclusion, we have described a metabolic signature of increased risk for future type 2 diabetes in large population-based cohorts of young adults with long follow-up. Metabolic aberrations were observed across multiple biological pathways, including inflammation, fatty acid balance and aspects of lipoprotein metabolism. Our results extend the evidence of amino acid alterations as strong predictors of type 2 diabetes to young adults. If branched-chain amino acids, MUFAs or *n*-6 fatty acids are proven to be causal in the pathogenesis of type 2 diabetes, then interventions aimed at altering the circulating levels may be beneficial in early adulthood. The detailed metabolic profiling was shown to capture aspects of the development of insulin resistance and post-load hyperglycaemia, which are missed by fasting glucose and other risk markers used in primary care settings. These results support the possibility that screening aided by detailed metabolic profiling could help targeting interventions for type 2 diabetes prevention in young adults.

## Electronic supplementary material

ESM (PDF 3.20 mb)

ESM Tables 2 and 3 (XLSX 528 kb)

## Data Availability

The individual-level data from DILGOM and FINRISK are available from THL Biobank (https://thl.fi/en/web/thl-biobank). Individual-level data for NFBC 1966 can be applied for from University of Oulu Infrastructure for Population Studies (www.oulu.fi/nfbc/) and for YFS by request to University of Turku Research Centre of Applied and Preventive Cardiovascular Medicine (youngfinnsstudy.utu.fi).

## Abbreviations

DILGOM: Dietary, Lifestyle, and Genetic Determinants of Obesity and Metabolic Syndrome Study
GlycA: Glycoprotein acetyls
IDL: Intermediate-density lipoprotein
MUFA: Monounsaturated fatty acids
NFBC: Northern Finland Birth Cohort
YFS: Young Finns Study
