# Supplementary material for: Circulating metabolites and the risk of type 2 diabetes: a prospective study of 11,896 young adults from four Finnish cohorts
Source: Diabetologia. 2019 Oct 4;62(12):2298–309. doi: 10.1007/s00125-019-05001-w (PMC6861432; doi:10.1007/s00125-019-05001-w)
Supplement: Supplementary file 1 — (PDF 3.20 mb) [file 125_2019_5001_MOESM1_ESM.pdf]

Online-only ESM material

Ahola-Olli *et al*, Circulating metabolites and the risk of type 2 diabetes: a prospective study of 11,896 young adults from four Finnish cohorts.

ESM Methods: Cohort descriptions and type 2 diabetes status assessment.
ESM Table 1: Mean (SD) concentrations of 229 metabolic metabolites analyzed.
ESM Table 2: Tabulation of all biomarker results in the figures (online spreadsheet).
ESM Table 3: Type 2 diabetes prediction models with standard clinical risk factors compared to biomarker enhanced model.
ESM Table 4: Regression models for risk of future type 2 diabetes.
ESM Table 5: Risk discrimination of three prediction models for risk of type 2 diabetes, assessed among 5,271 individuals aged 31.
ESM Fig. 1: Overview of study cohorts and participant inclusion.
ESM Fig. 2: Relation of 125 metabolic measures (not shown in main paper) to risk of future type 2 diabetes.
ESM Fig. 3: Consistency of biomarkers to the risk of future diabetes across the four cohorts.
ESM Fig. 4: Biomarkers for the risk of future diabetes assessed separately for men and women.
ESM Fig. 5: Biomarker associations with diabetes risk after additional adjustment for HOMA-IR and without adjustment for BMI.
ESM Fig. 6: Correlations of overall biomarker association patterns for fasting glucose, 2h glucose and HOMA-IR at follow-up.
ESM Fig. 7: Biomarker associations with incident impaired fasting glucose compared with incident type 2 diabetes.
ESM Fig. 8: Biomarkers for the risk of future diabetes compared to cross-sectional associations with BMI, HOMA-IR and fasting glucose.
ESM Fig. 9: Refinement of absolute risk for type 2 diabetes by including multi-metabolite score into prediction model.

ESM Methods: Cohort descriptions and type 2 diabetes status assessment.

The Cardiovascular Risk in Young Finns Study

The Cardiovascular Risk in Young Finns Study (YFS) is an ongoing longitudinal multi-center follow-up of 3,596 Finnish individuals randomly chosen from the cities of Turku, Tampere, Helsinki, Oulu, and Kuopio, in addition to their rural surroundings (http://youngfinnsstudy.utu.fi)(1). The first cross-sectional survey was conducted in 1980 and the follow-up surveys concerning whole study population were conducted in 1983, 1986, 2001, 2007, and 2011. NMR metabolomics has been performed from n~2,200 serum samples drawn from all participants attending the 2001, 2007, and 2011 follow-up visits after overnight fasting using the Nightingale Health platform (2016 quantification version) (2). The metabolic measures acquired from serum samples from the 2001 collection (n=2,246 available) form the baseline data for the present study. Pregnant women were omitted from the analyses (n=78). Individuals with prevalent diabetes at the 2001-baseline were excluded from the analyses, based on self-reported diabetes (type 1 and type 2), use of anti-diabetic medications and fasting plasma glucose ≥ 7.0 mmol/l (n=23).

Outcome information on diabetes status at follow-up was based on a combination of fasting plasma glucose ≥7 mmol/L at either of the 2007 and the 2011 surveys, or if they reported having been given a type 2 diabetes diagnosis by a physician. Individuals whose HbA_{1c} was ≥6.5% (48 mmol/mol) at the 2011 follow-up or who reported taking glucose-lowering medication at the 2007 or 2011 follow-up were also classified as having type 2 diabetes. To complement the information on diabetes outcome status from the follow-up surveys, type 2 diabetes diagnoses were obtained from the National Social Insurance Institution Drug Reimbursement Registry and nationwide hospital discharge registries, as detailed in the paragraph of registry tracking of diabetes incidence (3). All participants gave written informed consent, and the study was approved by the local ethics committees of the study sites.

FINRISK-1997

FINRISK is a series of population-based health examination surveys to evaluate the cardiovascular risk status in Finnish population (4). The first survey was done in 1972 in provinces of North Karelia and Northern Savo. Subsequently, the FINRISK surveys have been expanded to surrounding regions of five Finnish cities: Turku, Loimaa, Helsinki, Vantaa, and Oulu. The surveys have been undertaken every five years and a new randomly chosen sample representative of population aged 25-74 (since 1997) has been invited to the surveys every time. Each study visit has contained questionnaires, physical examination and venous blood samples. Data for the present study stems from the FINRISK 1997 survey, in which 8,444 individuals participated. Serum samples for NMR metabolomics were available for 7,602 individuals, and measured using the Nightingale Health platform (2016 quantification version)(2). The median fasting time before the blood samples were drawn was 5 hours (interquartile range 4-6 hours). Individuals over 45 years at baseline were excluded from the analysis; out of 3516 participants aged 24–45, metabolomics data were available for 3210.

We used several data sources to ascertain exclusion of prevalent diabetes at baseline for (n=75): a) self-report of doctor-diagnosed diabetes or impaired glucose tolerance in the questionnaire, b) blood glucose ≥7.0 mmol/L at baseline, and c) the national drug reimbursement records and the National Hospital Discharge Register were checked for reimbursements of purchases of hypoglycemic drugs or hospitalizations with diabetes as the main or an additional diagnosis. If any of these sources was positive, the person was considered as having prevalent diabetes and was excluded from the analyses.

For tracking information about incidence of type 2 diabetes, we used information on National Social Insurance Institution Drug Reimbursement Registry and nation-wide hospital discharge register as described previously and detailed further below (5). The register data used in this study

included diagnoses made between spring 1997 and December 2012 (15-year follow-up). No laboratory data were available for FINRISK1997 to confirm incident diabetes status.
All participants gave written informed consent and the FINRISK study was approved by the ethical committee of the National Public Health Institute, Helsinki, Finland.

Dietary, Lifestyle, and Genetic determinants of Obesity and Metabolic syndrome study

The Dietary, Lifestyle, and Genetic determinants of Obesity and Metabolic syndrome (DILGOM) study is an extension of the FINRISK 2007 survey, focused on obesity and metabolic syndrome (6). All FINRISK 2007 participants were invited for a re-survey, and 84% participated(n=5,024). DILGOM study participants living in Southwest Finland were re-examined in 2014. They completed questionnaires, gave blood samples and underwent physical examination. Individuals living in the Oulu province, North Karelia, or Northern Savo completed a questionnaire but did not participate in physical examination nor gave blood samples.
In the present study, NMR metabolomics data from 4,816 individuals were available from fasting blood samples. Individuals over 45 years at baseline and pregnant women were excluded from the analysis, leaving 1,488 individuals for the analyses. Individuals with prevalent diabetes in the 2007 survey were excluded from the analyses, based on self-reported diabetes, use of anti-diabetic medications, and fasting plasma glucose ≥ 7.0 mmol/l or 2h glucose ≥11.0 (n=67). This information was further complemented from the nationwide drug-reimbursement and hospital registries as for FINRISK 1997 participants.
For incident diabetes, we used register-based diagnosis as described above for FINRISK 1997, follow-up time until end of 2014 (7.8 years). In addition, for participants living in southwestern Finland we had data fasting glucose available at the 7-year re-survey and individuals with fasting glucose ≥ 7.0 mmol/l were assigned as diabetes cases.
The Coordinating Ethics Committee of the Hospital District of Helsinki and Uusimaa has approved the FINRISK 2007 study and the DILGOM extension.

Northern Finland Birth Cohort of 1966

The Northern Finland Birth Cohort (NFBC) of 1966 is a longitudinal follow-up study of 12,058 children born into the cohort, comprising 96% of all births during 1966 in the region (www.oulu.fi/NFBC) (7). The participants were enrolled to the study in 1965 when their mothers were on their 24th gestational week. Majority of the children were born in 1966, excluding a small subset born in the end of 1965 or early 1967. Follow-up surveys have been arranged when the participants were 0-1, 14, 31 or 46 years of age. Data collection in 1997 included clinical examination and serum sampling at age 30–32 for 6,007 individuals, which form the baseline for the present study (2, 8). Attendees in the 1997 field study (70% of those invited for survey; 52% of original cohort) were representative of the original cohort.
NMR-based metabolomics (Nightingale Health Ltd, 2016 quantification version) was measured from 5,709 individuals with serum sample available, of which 96% were fasting samples. Individuals with prevalent diabetes at baseline were excluded from analyses. Information on diabetes outcome status was based on fasting glucose (≥ 7.0 mmol/l) and OGTT (2-hour value ≥ 11.0 mmol/l) assessed at the 2012 follow-up when the participants were 46 years old (data available for 3306 individuals). The diabetes outcome status from OGTT was complemented using nationwide reimbursement registries on prescription medication and hospital registry data for all participants with metabolomics at 31-year baseline as detailed below.
Informed written consent was obtained from all participants. The research protocols were approved by the Ethics Committees s of University of Oulu and that of Northern Ostrobotnia Hospital District, Finland.

Registry information on incidence of type 2 diabetes

Three data sources were used to identify cases of incident diabetes during the follow-up: 1) Record linkage of the study participants with the National Social Insurance Institution Drug Reimbursement Registry on the basis of the personal identification code unique to each individual in the country. The Social Insurance Institute keeps a nation-wide register of persons entitled to these reimbursements. 2) Record linkage with the National Hospital Discharge Register, which includes all hospitalizations in Finland (main diagnosis and up to four additional diagnoses). We checked whether diabetes (ICD-10 code E10-E14) was listed as any of the diagnoses for a hospitalization during the follow-up. 3) Record linkage with the National Causes-of-Death Register, which includes all deaths of permanent residents of Finland. We checked whether diabetes (ICD-10 code E10-E14) was mentioned as any of the causes of death (underlying cause of death, direct cause of death, or the contributing causes of death). If diabetes was found in any of these data sources, the person was considered to have incident diabetes. The date when the diabetes diagnosis first appeared was taken as the date of onset of diabetes. These procedures identify all cases of diabetes that were treated with hypoglycemic medications or hospitalized or who died during the follow-up. However, diabetic patients treated with diet only, who were not hospitalized and did not die, were not identified by these procedures (5).

ESM References

1. Raitakari OT, Juonala M, Ronnemaa T, Keltikangas-Jarvinen L, Rasanen L, Pietikainen M, et al. Cohort profile: the cardiovascular risk in Young Finns Study. *Int J Epidemiol*. 2008;37(6):1220-6.
2. Würtz P, Wang Q, Kangas AJ, Richmond RC, Skarp J, Tiainen M, et al. Metabolic signatures of adiposity in young adults: Mendelian randomization analysis and effects of weight change. *PLoS Med*. 2014;11(12):e1001765.
3. Pitkänen N, Juonala M, Ronnemaa T, Sabin MA, Hutri-Kahonen N, Kahonen M, et al. Role of Conventional Childhood Risk Factors Versus Genetic Risk in the Development of Type 2 Diabetes and Impaired Fasting Glucose in Adulthood: The Cardiovascular Risk in Young Finns Study. *Diabetes Care*. 2016;39(8):1393-9.
4. Borodulin K, Tolonen H, Jousilahti P, Jula A, Juolevi A, Koskinen S, et al. Cohort Profile: The National FINRISK Study. *Int J Epidemiol*. 2017. doi: 10.1093/ije/dyx239
5. Salomaa V, Havulinna A, Saarela O, Zeller T, Jousilahti P, Jula A, et al. Thirty-one novel biomarkers as predictors for clinically incident diabetes. *PLoS One*. 2010;5(4):e10100.
6. Konttinen H, Llewellyn C, Silventoinen K, Joensuu A, Mannisto S, Salomaa V, et al. Genetic predisposition to obesity, restrained eating and changes in body weight: a population-based prospective study. *Int J Obes (Lond)*. 2017.
7. Järvelin MR, Sovio U, King V, Lauren L, Xu B, McCarthy MI, et al. Early life factors and blood pressure at age 31 years in the 1966 northern Finland birth cohort. *Hypertension*. 2004;44(6):838-46.
8. Würtz P, Makinen VP, Soininen P, Kangas AJ, Tukiainen T, Kettunen J, et al. Metabolic signatures of insulin resistance in 7,098 young adults. *Diabetes*. 2012;61(6):1372-80.
9. Pencina MJ, D'Agostino RB, Sr., Steyerberg EW. Extensions of net reclassification improvement calculations to measure usefulness of new biomarkers. *Stat Med*. 2011;30(1):11-21.
10. Würtz P, Havulinna AS, Soininen P, Tynkkynen T, Prieto-Merino D, Tillin T, et al. Metabolite profiling and cardiovascular event risk: a prospective study of 3 population-based cohorts. *Circulation*. 2015;131(9):774-85.

ESM Table 1. Mean (SD) concentrations of 229 metabolic measure analyzed and their odds ratios for incident type 2 diabetes

Metabolic measure	Units	Mean concen-tration	Standard deviation (SD)	Odds ratio per 1-SD	95% CI	P-value
Isoleucine	mmol/l	0.056	0.018	1.329	1.205–1.466	1E-8
Leucine	mmol/l	0.084	0.021	1.331	1.205–1.469	2E-8
Valine	mmol/l	0.20	0.048	1.200	1.076–1.339	0.001
Phenylalanine	mmol/l	0.081	0.013	1.312	1.180–1.458	5E-7
Tyrosine	mmol/l	0.054	0.013	1.183	1.064–1.315	0.002
Alanine	mmol/l	0.41	0.068	1.130	1.016–1.256	0.02
Glutamine	mmol/l	0.51	0.084	0.842	0.752–0.943	0.003
Glycine	mmol/l	0.30	0.063	0.926	0.820–1.045	0.21
Histidine	mmol/l	0.071	0.011	1.048	0.941–1.168	0.39
Lactate	mmol/l	1.38	0.38	1.126	1.015–1.250	0.02
Pyruvate	mmol/l	0.083	0.027	1.084	0.987–1.192	0.09
Glycerol	mmol/l	0.11	0.058	1.218	1.103–1.346	1E-4
Acetoacetate	mmol/l	0.062	0.049	1.085	0.977–1.204	0.13
beta-hydroxybutyrate	mmol/l	0.18	0.14	1.097	0.983–1.224	0.10
Glycoprotein Acetyls	mmol/l	1.34	0.23	1.368	1.236–1.514	2E-9
Creatinine	mmol/l	0.062	0.013	0.927	0.810–1.061	0.27
Albumin	signal area	0.10	0.011	1.032	0.925–1.152	0.57
Acetate	mmol/l	0.048	0.033	0.871	0.697–1.088	0.22
Citrate	mmol/l	0.11	0.019	0.915	0.816–1.026	0.13
Total fatty acids	mmol/l	12.03	2.95	1.229	1.107–1.364	1E-4
Saturated fatty acids, relative to total fatty acids	%	36.90	2.29	1.140	1.027–1.265	0.01
Monounsaturated fatty acids, relative to total fatty acids	%	25.60	2.86	1.317	1.176–1.475	2E-6
Polyunsaturated fatty acids, relative to total fatty acids	%	37.52	3.62	0.768	0.698–0.845	7E-8
Omega-6 fatty acids, relative to total fatty acids	%	33.02	3.24	0.754	0.686–0.829	6E-9
Linoleic acid, relative to total fatty acids	%	26.95	3.52	0.751	0.681–0.828	7E-9
Arachidonic acid, relative to total fatty acids	%	6.07	1.13	0.953	0.858–1.060	0.38
Omega-3 fatty acids, relative to total fatty acids	%	4.49	1.09	0.974	0.878–1.082	0.63
Docosahexaenoic acid, relative to total fatty acids	%	1.60	0.56	1.060	0.956–1.176	0.27
Unsaturation degree	-	1.19	0.061	0.847	0.765–0.939	0.002
Sphingomyelins	mmol/l	0.52	0.10	1.060	0.950–1.181	0.30
Total cholines	mmol/l	2.49	0.51	1.060	0.951–1.182	0.29
Phosphatidylcholines	mmol/l	2.05	0.44	1.082	0.971–1.206	0.15
Total phosphoglycerides	mmol/l	2.05	0.48	1.083	0.973–1.206	0.14

Ratio of triacylglycerols to phosphoglycerides	-	0.56	0.21	1.425	1.292–1.573	2E-12
Total serum cholesterol	mmol/l	4.82	1.07	1.061	0.951–1.183	0.29
VLDL cholesterol	mol/l	0.76	0.33	1.310	1.174–1.463	1E-6
LDL cholesterol	mmol/l	1.73	0.57	1.095	0.980–1.223	0.11
HDL cholesterol	mmol/l	1.56	0.38	0.747	0.658–0.847	6E-6
Total triacylglycerols	mmol/l	1.16	0.62	1.381	1.249–1.527	3E-10
VLDL triacylglycerols	mmol/l	0.70	0.49	1.378	1.249–1.521	2E-10
LDL triacylglycerols	mmol/l	0.19	0.10	1.257	1.143–1.383	3E-6
HDL triacylglycerols	mmol/l	0.15	0.05	1.154	1.042–1.279	0.006
Apolipoprotein B	g/l	0.90	0.23	1.252	1.123–1.396	5E-5
Apolipoprotein A1	g/l	1.59	0.23	0.872	0.773–0.984	0.03
ApoB ratio to ApoA1	-	0.58	0.16	1.403	1.248–1.578	2E-8
VLDL particle size, diameter	nm	35.79	1.33	1.325	1.193–1.471	1E-7
LDL particle size, diameter	nm	23.66	0.17	0.942	0.842–1.054	0.30
HDL particle size, diameter	nm	10.07	0.30	0.622	0.541–0.716	4E-11
XXL VLDL cholesterol	mmol/l	0.005	0.006	1.200	1.108–1.299	8E-6
XL VLDL cholesterol	mmol/l	0.013	0.016	1.221	1.127–1.323	1E-6
L VLDL cholesterol	mmol/l	0.051	0.053	1.265	1.161–1.377	7E-8
M VLDL cholesterol	mmol/l	0.15	0.09	1.285	1.167–1.415	3E-7
S VLDL cholesterol	mmol/l	0.24	0.10	1.241	1.112–1.385	1E-4
XS VLDL cholesterol	mmol/l	0.30	0.10	1.139	1.023–1.269	0.02
IDL cholesterol	mmol/l	0.77	0.23	1.075	0.965–1.198	0.19
L LDL cholesterol	mmol/l	0.92	0.29	1.094	0.981–1.220	0.11
M LDL cholesterol	mmol/l	0.50	0.18	1.100	0.987–1.226	0.09
S LDL cholesterol	mmol/l	0.31	0.11	1.089	0.978–1.212	0.12
XL HDL cholesterol	mmol/l	0.28	0.15	0.780	0.690–0.882	8E-5
L HDL cholesterol	mmol/l	0.41	0.21	0.629	0.545–0.725	2E-10
M HDL cholesterol	mmol/l	0.43	0.15	0.901	0.807–1.005	0.06
S HDL cholesterol	mmol/l	0.45	0.10	1.032	0.931–1.144	0.55
XXL VLDL triacylglycerols	mmol/l	0.018	0.020	1.207	1.118–1.302	1E-6
XL VLDL triacylglycerols	mmol/l	0.030	0.041	1.240	1.149–1.337	3E-8
L VLDL triacylglycerols	mmol/l	0.10	0.12	1.292	1.189–1.403	1E-9
M VLDL triacylglycerols	mmol/l	0.22	0.17	1.329	1.214–1.456	9E-10
S VLDL triacylglycerols	mmol/l	0.21	0.11	1.349	1.223–1.488	2E-9
XS VLDL triacylglycerols	mmol/l	0.11	0.05	1.316	1.197–1.446	1E-8
IDL triacylglycerols	mmol/l	0.12	0.06	1.257	1.147–1.378	1E-6
L LDL triacylglycerols	mmol/l	0.11	0.05	1.243	1.132–1.365	5E-6
M LDL triacylglycerols	mmol/l	0.051	0.028	1.229	1.120–1.349	1E-5
S LDL triacylglycerols	mmol/l	0.031	0.017	1.264	1.154–1.384	4E-7
XL HDL triacylglycerols	mmol/l	0.017	0.010	1.028	0.924–1.143	0.61
L HDL triacylglycerols	mmol/l	0.036	0.019	0.843	0.741–0.957	0.009

M HDL triacylglycerols	mmol/l	0.045	0.018	1.191	1.081–1.313	4E-4
S HDL triacylglycerols	mmol/l	0.049	0.020	1.299	1.179–1.432	1E-7
XXL VLDL cholesterol, ratio to total lipids	%	19.04	5.21	0.929	0.823–1.049	0.24
XL VLDL cholesterol, ratio to total lipids	%	29.13	11.80	0.735	0.658–0.820	4E-8
L VLDL cholesterol, ratio to total lipids	%	28.23	8.26	0.792	0.716–0.875	5E-6
M VLDL cholesterol, ratio to total lipids	%	32.97	6.47	0.759	0.678–0.848	1E-6
S VLDL cholesterol, ratio to total lipids	%	41.54	6.49	0.797	0.717–0.886	2E-5
XS VLDL cholesterol, ratio to total lipids	%	52.01	5.32	0.817	0.745–0.896	2E-5
IDL cholesterol, ratio to total lipids	%	63.14	3.10	0.832	0.760–0.911	7E-5
L LDL cholesterol, ratio to total lipids	%	66.63	3.06	0.866	0.784–0.956	0.004
M LDL cholesterol, ratio to total lipids	%	65.13	4.77	0.897	0.814–0.988	0.03
S LDL cholesterol, ratio to total lipids	%	62.16	5.08	0.924	0.845–1.009	0.08
XL HDL cholesterol, ratio to total lipids	%	48.95	8.30	1.275	1.130–1.438	8E-5
L HDL cholesterol, ratio to total lipids	%	47.20	5.08	0.848	0.782–0.921	8E-5
M HDL cholesterol, ratio to total lipids	%	47.30	5.30	0.848	0.781–0.922	1E-4
S HDL cholesterol, ratio to total lipids	%	41.00	4.49	0.932	0.852–1.020	0.13
XXL VLDL triacylglycerols, ratio to total lipids	%	70.67	6.17	1.082	0.950–1.233	0.24
XL VLDL triacylglycerols, ratio to total lipids	%	53.20	13.70	1.502	1.231–1.831	6E-5
L VLDL triacylglycerols, ratio to total lipids	%	52.98	8.98	1.327	1.122–1.570	0.001
M VLDL triacylglycerols, ratio to total lipids	%	45.73	7.17	1.409	1.208–1.644	1E-5
S VLDL triacylglycerols, ratio to total lipids	%	35.09	6.33	1.371	1.201–1.565	3E-6
XS VLDL triacylglycerols, ratio to total lipids	%	19.12	4.81	1.338	1.195–1.499	5E-7
IDL triacylglycerols, ratio to total lipids	%	10.13	3.07	1.302	1.173–1.446	8E-7
L LDL triacylglycerols, ratio to total lipids	%	8.03	2.77	1.265	1.139–1.406	1E-5
M LDL triacylglycerols, ratio to total lipids	%	6.72	2.81	1.237	1.108–1.382	2E-4
S LDL triacylglycerols, ratio to total lipids	%	6.27	2.64	1.286	1.155–1.432	4E-6
XL HDL triacylglycerols, ratio to total lipids	%	3.78	3.54	1.218	1.101–1.348	1E-4

L HDL triacylglycerols, ratio to total lipids	%	4.51	2.37	1.153	1.036–1.283	0.009
M HDL triacylglycerols, ratio to total lipids	%	5.36	2.64	1.293	1.155–1.447	8E-6
S HDL triacylglycerols, ratio to total lipids	%	4.58	2.03	1.311	1.169–1.469	3E-6
HDL2 cholesterol	mmol/l	1.04	0.36	0.733	0.647–0.831	1E-6
HDL3 cholesterol	mmol/l	0.52	0.05	0.981	0.883–1.090	0.72
Esterified cholesterol	mmol/l	3.43	0.77	1.057	0.948–1.179	0.32
Free cholesterol	mmol/l	1.39	0.30	1.074	0.964–1.196	0.19
Remnant cholesterol	mmol/l	1.53	0.51	1.232	1.099–1.380	3E-4
Saturated fatty acids	mmol/l	4.46	1.22	1.241	1.121–1.375	3E-5
Monounsaturated fatty acids	mmol/l	3.12	1.06	1.290	1.164–1.429	1E-6
Polyunsaturated fatty acids	mmol/l	4.45	0.86	1.100	0.988–1.224	0.08
Omega-6 fatty acids	mmol/l	3.91	0.75	1.084	0.974–1.207	0.14
Linoleic acid	mmol/l	3.18	0.59	1.051	0.944–1.171	0.36
Arachidonic acid	mmol/l	0.73	0.23	1.190	1.072–1.320	0.001
Omega-3 fatty acids	mmol/l	0.54	0.17	1.136	1.029–1.254	0.01
Docosahexaenoic acid	mmol/l	0.19	0.07	1.174	1.066–1.292	0.001
XXL VLDL lipid concentration	mmol/l	0.027	0.029	1.211	1.121–1.309	1E-6
XL VLDL lipid concentration	mmol/l	0.052	0.067	1.250	1.155–1.353	3E-8
L VLDL lipid concentration	mmol/l	0.19	0.21	1.312	1.201–1.433	2E-9
M VLDL lipid concentration	mmol/l	0.47	0.32	1.348	1.221–1.488	3E-9
S VLDL lipid concentration	mmol/l	0.58	0.25	1.353	1.212–1.510	7E-8
XS VLDL lipid concentration	mmol/l	0.57	0.18	1.223	1.099–1.360	2E-4
IDL lipid concentration	mmol/l	1.21	0.34	1.119	1.004–1.247	0.04
L LDL lipid concentration	mmol/l	1.37	0.40	1.127	1.010–1.258	0.03
M LDL lipid concentration	mmol/l	0.76	0.24	1.143	1.025–1.275	0.02
S LDL lipid concentration	mmol/l	0.49	0.15	1.140	1.023–1.269	0.02
XL HDL lipid concentration	mmol/l	0.56	0.29	0.717	0.630–0.815	4E-7
L HDL lipid concentration	mmol/l	0.84	0.39	0.671	0.591–0.763	1E-9
M HDL lipid concentration	mmol/l	0.89	0.26	0.933	0.840–1.037	0.20
S HDL lipid concentration	mmol/l	1.09	0.18	1.076	0.973–1.189	0.15
XXL VLDL particle concentration	mol/l	1.24E-10	1.33E-10	1.194	1.106–1.288	5E-6
XL VLDL particle concentration	mol/l	5.20E-10	6.79E-10	1.217	1.129–1.312	3E-7
L VLDL particle concentration	mol/l	3.25E-9	3.63E-9	1.245	1.153–1.345	3E-8
M VLDL particle concentration	mol/l	1.39E-8	9.52E-9	1.277	1.174–1.390	1E-8
S VLDL particle concentration	mol/l	2.91E-8	1.29E-8	1.311	1.189–1.445	6E-8

XS VLDL particle concentration	mol/l	4.44E-8	1.45E-8	1.218	1.104–1.344	9E-5
IDL particle concentration	mol/l	1.20E-7	3.37E-8	1.125	1.016–1.247	0.02
L LDL particle concentration	mol/l	1.92E-7	5.64E-8	1.132	1.021–1.254	0.02
M LDL particle concentration	mol/l	1.49E-7	4.81E-8	1.145	1.033–1.269	0.01
S LDL particle concentration	mol/l	1.72E-7	5.25E-8	1.146	1.034–1.270	0.009
XL HDL particle concentration	mol/l	5.47E-7	2.83E-7	0.677	0.585–0.783	2E-7
L HDL particle concentration	mol/l	1.34E-6	6.06E-7	0.646	0.556–0.751	1E-8
M HDL particle concentration	mol/l	2.09E-6	6.02E-7	0.959	0.860–1.069	0.45
S HDL particle concentration	mol/l	4.91E-6	8.12E-7	1.109	1.004–1.226	0.04
XXL VLDL cholesteryl esters	mmol/l	3.29E-3	3.43E-3	1.191	1.096–1.294	4E-5
XL VLDL cholesteryl esters	mmol/l	0.0072	0.0085	1.214	1.120–1.316	2E-6
L VLDL cholesteryl esters	mmol/l	0.030	0.028	1.247	1.142–1.361	9E-7
M VLDL cholesteryl esters	mmol/l	0.095	0.052	1.244	1.128–1.372	1E-5
S VLDL cholesteryl esters	mmol/l	0.16	0.069	1.185	1.061–1.322	0.002
XS VLDL cholesteryl esters	mmol/l	0.21	0.072	1.131	1.015–1.260	0.03
IDL cholesteryl esters	mmol/l	0.55	0.16	1.084	0.973–1.208	0.14
L LDL cholesteryl esters	mmol/l	0.65	0.22	1.105	0.991–1.233	0.07
M LDL cholesteryl esters	mmol/l	0.35	0.15	1.094	0.981–1.219	0.11
S LDL cholesteryl esters	mmol/l	0.21	0.09	1.080	0.971–1.202	0.16
XL HDL cholesteryl esters	mmol/l	0.20	0.11	0.787	0.697–0.889	1E-4
L HDL cholesteryl esters	mmol/l	0.32	0.16	0.636	0.551–0.733	5E-10
M HDL cholesteryl esters	mmol/l	0.34	0.12	0.900	0.807–1.005	0.06
S HDL cholesteryl esters	mmol/l	0.33	0.08	1.018	0.919–1.129	0.73
XXL VLDL free cholesterol	mmol/l	0.0020	0.0024	1.199	1.111–1.293	3E-6
XL VLDL free cholesterol	mmol/l	0.006	0.007	1.219	1.127–1.319	8E-7
L VLDL free cholesterol	mmol/l	0.021	0.026	1.256	1.160–1.359	2E-8
M VLDL free cholesterol	mmol/l	0.055	0.041	1.294	1.184–1.413	1E-8
S VLDL free cholesterol	mmol/l	0.082	0.036	1.297	1.170–1.436	7E-7
XS VLDL free cholesterol	mmol/l	0.091	0.030	1.140	1.029–1.263	0.01
IDL free cholesterol	mmol/l	0.22	0.07	1.050	0.945–1.166	0.37
L LDL free cholesterol	mmol/l	0.26	0.07	1.060	0.953–1.178	0.28
M LDL free cholesterol	mmol/l	0.15	0.04	1.121	1.009–1.246	0.03
S LDL free cholesterol	mmol/l	0.092	0.022	1.116	1.005–1.241	0.04
XL HDL free cholesterol	mmol/l	0.075	0.039	0.754	0.659–0.863	4E-5
L HDL free cholesterol	mmol/l	0.087	0.049	0.587	0.501–0.687	4E-11
M HDL free cholesterol	mmol/l	0.082	0.030	0.924	0.826–1.034	0.17
S HDL free cholesterol	mmol/l	0.116	0.022	1.072	0.970–1.186	0.17

XXL VLDL phospholipids	mmol/l	0.0029	0.0035	1.184	1.096–1.279	2E-5
XL VLDL phospholipids	mmol/l	0.009	0.012	1.224	1.134–1.321	2E-7
L VLDL phospholipids	mmol/l	0.035	0.039	1.270	1.172–1.377	6E-9
M VLDL phospholipids	mmol/l	0.10	0.06	1.307	1.194–1.431	7E-9
S VLDL phospholipids	mmol/l	0.13	0.05	1.321	1.191–1.464	1E-7
XS VLDL phospholipids	mmol/l	0.16	0.05	1.158	1.046–1.281	0.005
IDL phospholipids	mmol/l	0.32	0.08	1.098	0.988–1.220	0.08
L LDL phospholipids	mmol/l	0.34	0.08	1.120	1.008–1.246	0.04
M LDL phospholipids	mmol/l	0.21	0.05	1.190	1.072–1.321	0.001
S LDL phospholipids	mmol/l	0.15	0.03	1.181	1.066–1.309	0.002
XL HDL phospholipids	mmol/l	0.26	0.14	0.635	0.549–0.733	7E-10
L HDL phospholipids	mmol/l	0.40	0.17	0.690	0.605–0.786	2E-8
M HDL phospholipids	mmol/l	0.41	0.11	0.957	0.860–1.064	0.41
S HDL phospholipids	mmol/l	0.59	0.11	1.065	0.964–1.176	0.21
XXL VLDL cholesteryl esters, ratio to total lipids	%	12.25	4.63	0.882	0.788–0.987	0.03
XL VLDL cholesteryl esters, ratio to total lipids	%	15.46	7.10	0.765	0.686–0.852	1E-6
L VLDL cholesteryl esters, ratio to total lipids	%	18.22	7.62	0.770	0.696–0.853	5E-7
M VLDL cholesteryl esters, ratio to total lipids	%	21.85	6.34	0.757	0.685–0.838	6E-8
S VLDL cholesteryl esters, ratio to total lipids	%	27.46	6.23	0.797	0.719–0.882	1E-5
XS VLDL cholesteryl esters, ratio to total lipids	%	36.09	5.33	0.851	0.775–0.934	7E-4
IDL cholesteryl esters, ratio to total lipids	%	45.15	2.76	0.901	0.820–0.990	0.03
L LDL cholesteryl esters, ratio to total lipids	%	47.16	3.26	0.962	0.856–1.080	0.51
M LDL cholesteryl esters, ratio to total lipids	%	44.88	6.55	0.969	0.876–1.071	0.54
S LDL cholesteryl esters, ratio to total lipids	%	42.89	6.58	0.970	0.885–1.064	0.52
XL HDL cholesteryl esters, ratio to total lipids	%	35.51	7.82	1.200	1.065–1.353	0.003
L HDL cholesteryl esters, ratio to total lipids	%	37.43	3.60	0.910	0.838–0.988	0.02
M HDL cholesteryl esters, ratio to total lipids	%	38.22	4.88	0.864	0.795–0.940	7E-4
S HDL cholesteryl esters, ratio to total lipids	%	30.35	5.06	0.931	0.854–1.014	0.10
XXL VLDL free cholesterol, ratio to total lipids	%	6.79	2.18	1.138	0.992–1.304	0.06
XL VLDL free cholesterol, ratio to total lipids	%	13.68	6.35	0.777	0.689–0.875	3E-5
L VLDL free cholesterol, ratio to total lipids	%	10.01	3.03	1.163	0.995–1.359	0.06
M VLDL free cholesterol, ratio to total lipids	%	11.12	1.65	1.219	1.044–1.422	0.01

S VLDL free cholesterol, ratio to total lipids	%	14.09	0.87	0.915	0.827–1.011	0.08
XS VLDL free cholesterol, ratio to total lipids	%	15.92	1.11	0.864	0.799–0.936	3E-4
IDL free cholesterol, ratio to total lipids	%	17.99	1.14	0.887	0.825–0.954	0.001
L LDL free cholesterol, ratio to total lipids	%	19.48	1.11	0.812	0.748–0.882	7E-7
M LDL free cholesterol, ratio to total lipids	%	20.25	2.34	0.849	0.753–0.958	0.008
S LDL free cholesterol, ratio to total lipids	%	19.27	2.03	0.861	0.768–0.966	0.01
XL HDL free cholesterol, ratio to total lipids	%	13.45	2.14	1.216	1.084–1.364	9E-4
L HDL free cholesterol, ratio to total lipids	%	9.78	1.90	0.771	0.710–0.837	7E-10
M HDL free cholesterol, ratio to total lipids	%	9.08	0.82	0.860	0.794–0.930	2E-4
S HDL free cholesterol, ratio to total lipids	%	10.64	0.84	0.975	0.880–1.079	0.62
XXL VLDL phospholipids, ratio to total lipids	%	10.30	2.46	0.999	0.883–1.130	0.99
XL VLDL phospholipids, ratio to total lipids	%	17.67	4.33	0.892	0.791–1.006	0.06
L VLDL phospholipids, ratio to total lipids	%	18.80	2.50	0.986	0.874–1.113	0.82
M VLDL phospholipids, ratio to total lipids	%	21.32	1.30	0.778	0.683–0.885	1E-4
S VLDL phospholipids, ratio to total lipids	%	23.38	1.67	0.837	0.748–0.936	0.002
XS VLDL phospholipids, ratio to total lipids	%	28.89	2.93	0.917	0.828–1.016	0.10
IDL phospholipids, ratio to total lipids	%	26.74	1.09	0.842	0.758–0.934	0.001
L LDL phospholipids, ratio to total lipids	%	25.34	1.69	0.905	0.807–1.014	0.09
M LDL phospholipids, ratio to total lipids	%	28.16	3.41	1.012	0.909–1.127	0.83
S LDL phospholipids, ratio to total lipids	%	31.58	4.10	0.990	0.887–1.105	0.86
XL HDL phospholipids, ratio to total lipids	%	47.28	9.15	0.843	0.782–0.910	1E-5
L HDL phospholipids, ratio to total lipids	%	48.29	4.46	1.169	1.061–1.288	0.002
M HDL phospholipids, ratio to total lipids	%	47.35	3.25	1.172	1.060–1.296	0.002
S HDL phospholipids, ratio to total lipids	%	54.43	4.35	0.980	0.886–1.084	0.69

Mean concentrations of the metabolic measures and standard deviations (SD) were averaged across the four cohorts. All 229 lipoprotein, lipid and metabolite measures were quantified using the Nightingale NMR metabolomics platform (Nightingale Health Ltd, Helsinki, Finland). The 14 lipoprotein subclass sizes were defined as follows: extremely large VLDL with particle diameters

from 75 nm upwards and a possible contribution of chylomicrons, five VLDL subclasses (average particle diameters of 64.0 nm, 53.6 nm, 44.5 nm, 36.8 nm, and 31.3 nm), IDL (28.6 nm), three LDL subclasses (25.5 nm, 23.0 nm, and 18.7 nm), and four HDL subclasses (14.3 nm, 12.1 nm, 10.9 nm, and 8.7 nm). The mean size for VLDL, LDL and HDL particles was calculated by weighting the corresponding subclass diameters with their particle concentrations. The lipoprotein composition measures were calculated as the ratio of a given lipid species (e.g. triacylglycerols) to the total lipid concentration within that particular lipoprotein subclass size.

ESM Table 2: Tabulation of all biomarker results in the figures (online spreadsheet).

ESM Table 3: Type 2 diabetes prediction models with standard clinical risk factors compared to biomarker enhanced model.

ESM Table 4. Regression models for risk of future type 2 diabetes.

	Basic clinical score			Extended clinical score			Multi-metabolite enhanced score		
**Variable**	**beta**	**SE**	**P**	**beta**	**SE**	**P**	**beta**	**SE**	**P**
Male sex	0.081	0.167	0.63	-0.091	0.184	0.62	-0.364	0.200	0.06
Age [years]	0.028	0.015	0.06	0.033	0.015	0.03	0.0286	0.0158	0.07
Body mass index [SD]	0.864	0.059	8E-48	0.681	0.072	3E-21	0.462	0.0740	4E-10
Fasting glucose [SD]	0.448	0.071	3E-10	0.343	0.068	4E-7	0.640	0.0719	5E-19
HDL cholesterol	-	-	-	-0.339	0.121	0.005	-	-	-
Triacylglycerols	-	-	-	-0.003	0.066	0.96	-	-	-
log_e(HOMA-IR)	-	-	-	0.323	0.087	0.0002	-	-	-
Large HDL free cholesterol	-	-	-	-	-	-	-0.474	0.117	5E-5
Phenylalanine	-	-	-	-	-	-	0.320	0.0802	6E-5
Large VLDL cholesterol ester %	-	-	-	-	-	-	-0.321	0.0847	2E-4

The weights (beta-coefficients in the multivariable logistic regression models) of three different risk scores for type 2 diabetes were derived by multivariable logistic regression using meta-analysis of three cohorts (YFS, FINRISK-1997, and DILGOM). The ‘basic clinical score’ was comprised of sex, baseline age, BMI and fasting glucose. An ‘extended clinical score’ was also examined, which included HDL cholesterol, triacylglycerols, and HOMA-IR in addition to the variables in the ‘basic clinical score’. These two models were compared to a ‘multi-metabolite enhanced score’ comprised of the variables in the ‘basic clinical score’ and additionally three variables metabolic biomarkers that entered the prediction model (phenylalanine, free cholesterol in large HDL, and cholesteryl esters to total lipids ratio within large VLDL). These three metabolic measures were selected among all clinical risk factors and the NMR measures using a forward step-wise process, which was meta-analyzed across the three derivation cohorts.

ESM Table 5. Risk discrimination of three prediction models for risk of type 2 diabetes, assessed among 5,271 individuals aged 31.

Model	C-statistic (95% CI)	Integrated discrimination improvement (IDI)	Continuous net reclassification improvement (NRI)
#1: Basic clinical score	0.729 (0.692-0.766)	-	-
#2: Extended clinical score	0.752 (0.717-0.786)	-	-
#3: Multi-metabolite enhanced score	0.764 (0.731-0.798);<br>P=0.0003 for #3 *vs* #1<br>P=0.13 for #3 *vs* #2	#3 *vs* 1:<br>1.32% net (P=7E-9)<br>#3 *vs* # 2:<br>0.51% net (P=0.03)	#3 *vs* 1<br>48.1% net (P=3E-11)<br>#3 *vs* 2<br>22.3% net (P=0.002)

The absolute risk estimates for assessing discrimination were derived by fitting the weighted sum of risk factors and metabolic biomarkers for each model ("the linear predictor") to the validation cohort (NFBC), and then calculated as

```
Absolute risk = 1/(1+exp(-(slope + scale(linear_predictor)))).
```

The slope and scaled beta-coefficient of the linear prediction was (-3.449; 0.704) for model #1, (-3.546;0.732) for model #2, and (-3.630;0.790) for model #3. The model weights from multivariable logistic as derived in meta-analysis of the 3 derivation cohorts are given in ESM Table 3. The DeLong method was used for calculating 95% CIs and P-value comparison of two C-statistics curves. IDI and NRI were calculated as previously described.[9,10]

ESM Fig.1. Overview of study cohorts and participant inclusion.

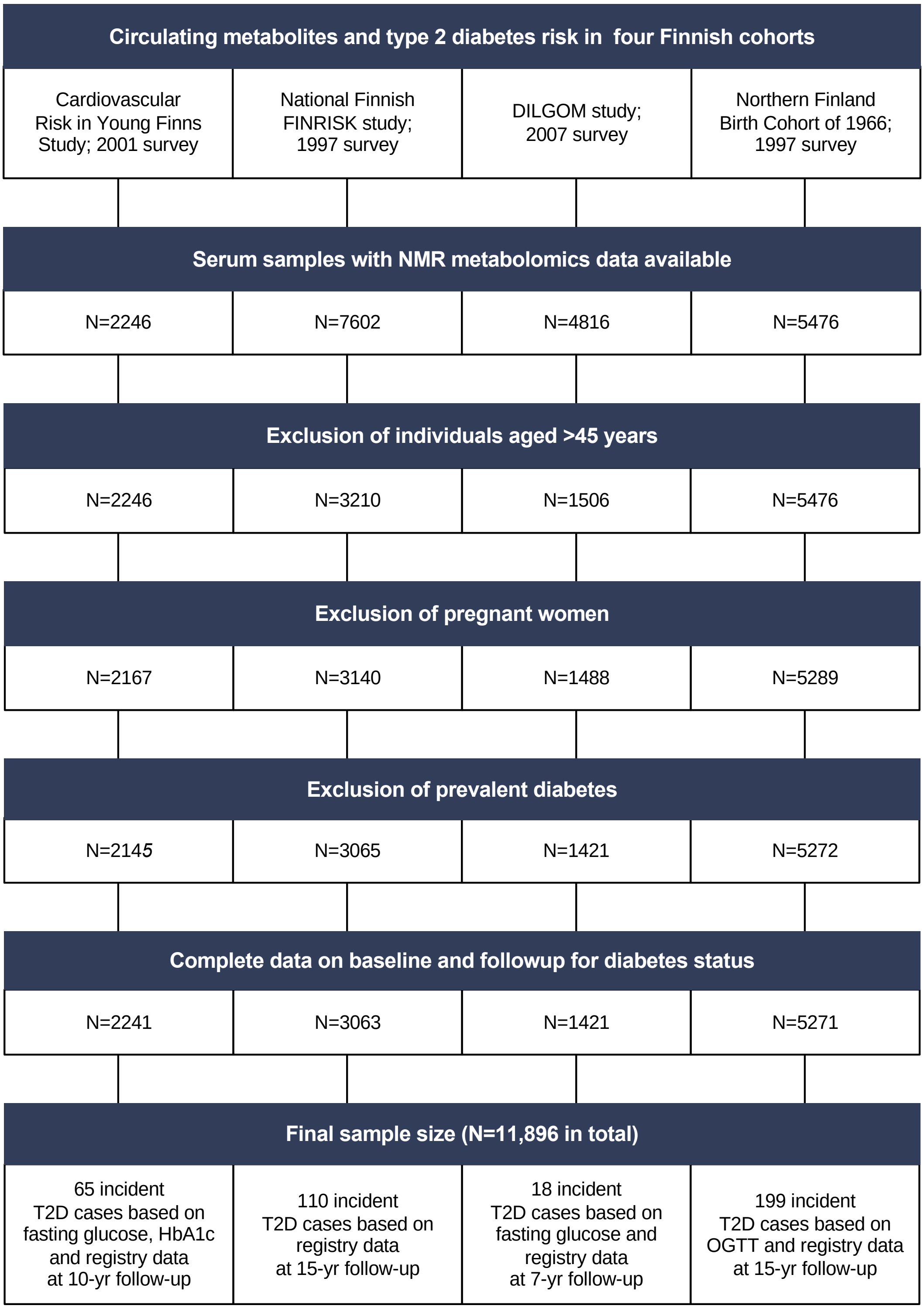

The flow-chart indicates numbers of participants in each cohort after specified exclusion criteria and number of cases of incident type 2 diabetes during 8–15 years of follow-up.

ESM Fig 2. Relation of 125 metabolic measures (not shown in main paper) to risk of future type 2 diabetes.

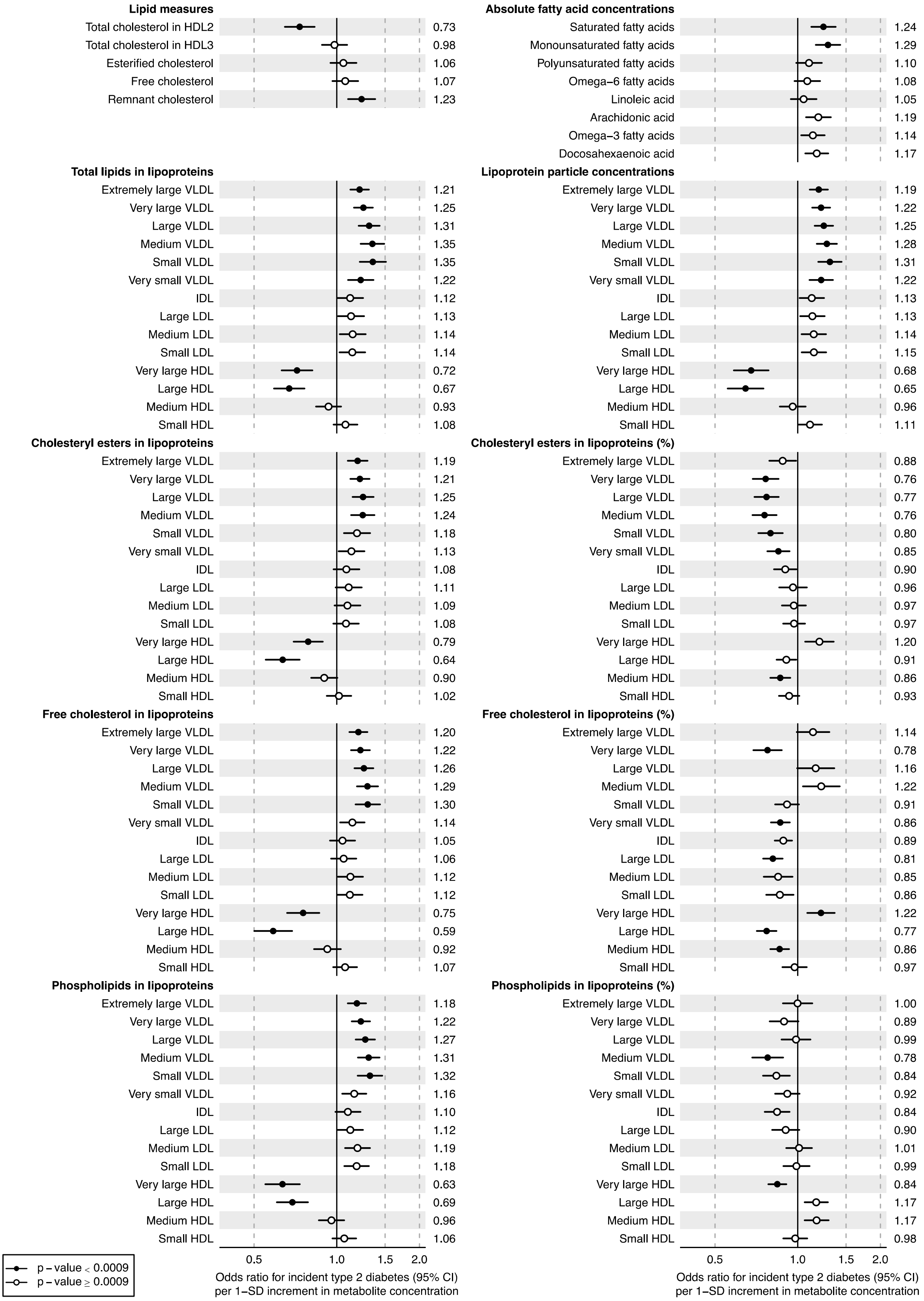

Values are odds ratios (95% confidence intervals) per 1-SD $\log_e$-transformed metabolite concentration. Odds ratios were adjusted for sex, baseline age, BMI, and fasting glucose. The results were meta-analyzed for 11,896 young adults from four prospective cohorts.

ESM Fig. 3. Consistency of biomarkers to the risk of future diabetes across the four cohorts.

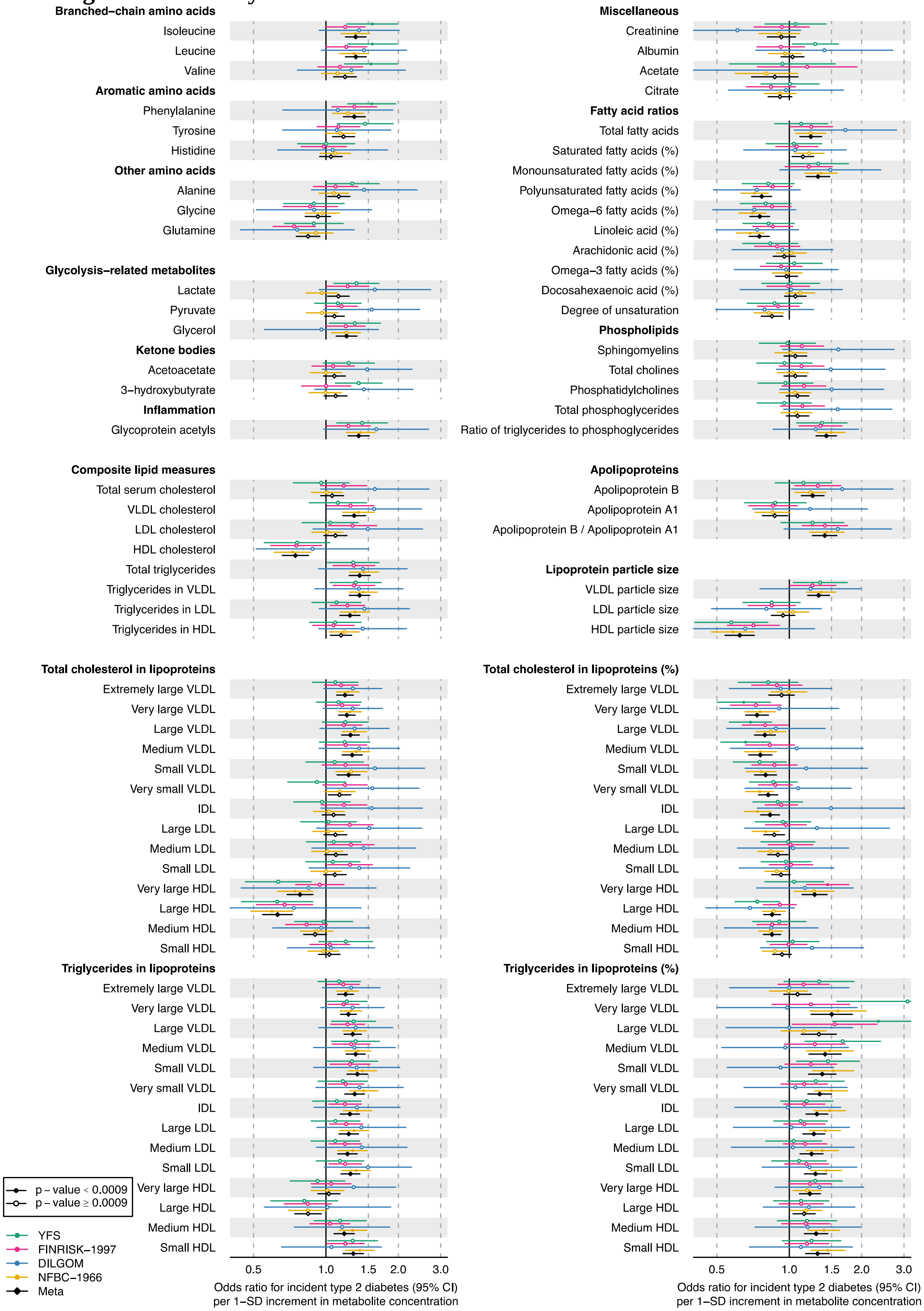

Values are odds ratios (95% confidence intervals) per 1-SD $\log_e$-transformed metabolite concentration. Odds ratios were adjusted for sex, baseline age, BMI, and fasting glucose. YFS, Cardiovascular risk in Young Finns Study; NFBC, Northern Finland Birth Cohort.

ESM Fig. 4. Biomarkers for the risk of future diabetes assessed separately for men and women.

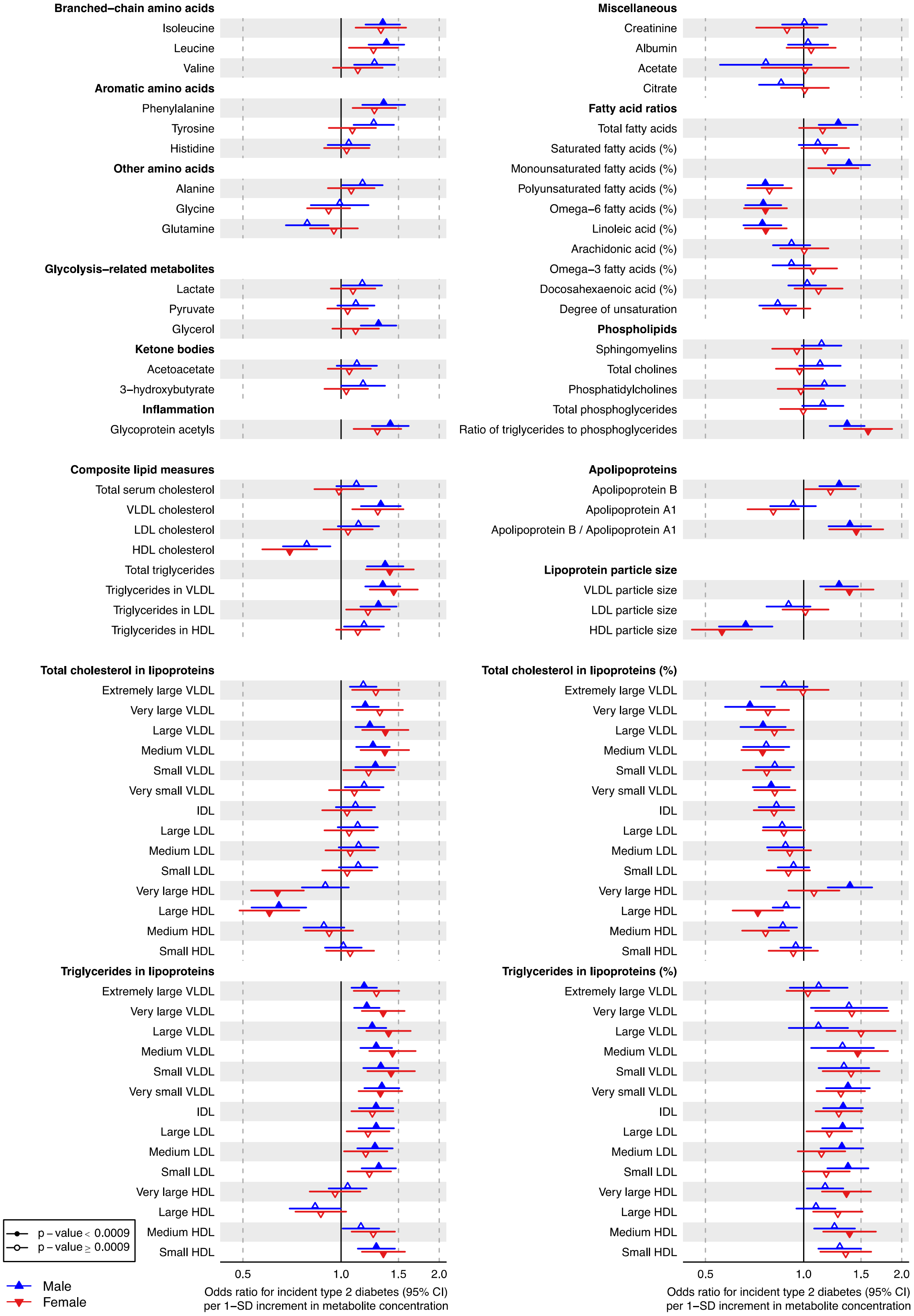

Values are odds ratios (95% confidence intervals) per 1-SD $\log_e$-transformed metabolite concentration. Odds ratios were adjusted for baseline age, BMI, and fasting glucose. Results were meta-analyzed across the 4 cohorts (5,696 men; 6,200 women).

ESM Fig. 5. Biomarker associations with diabetes risk after additional adjustment for HOMA-IR and without adjustment for BMI.

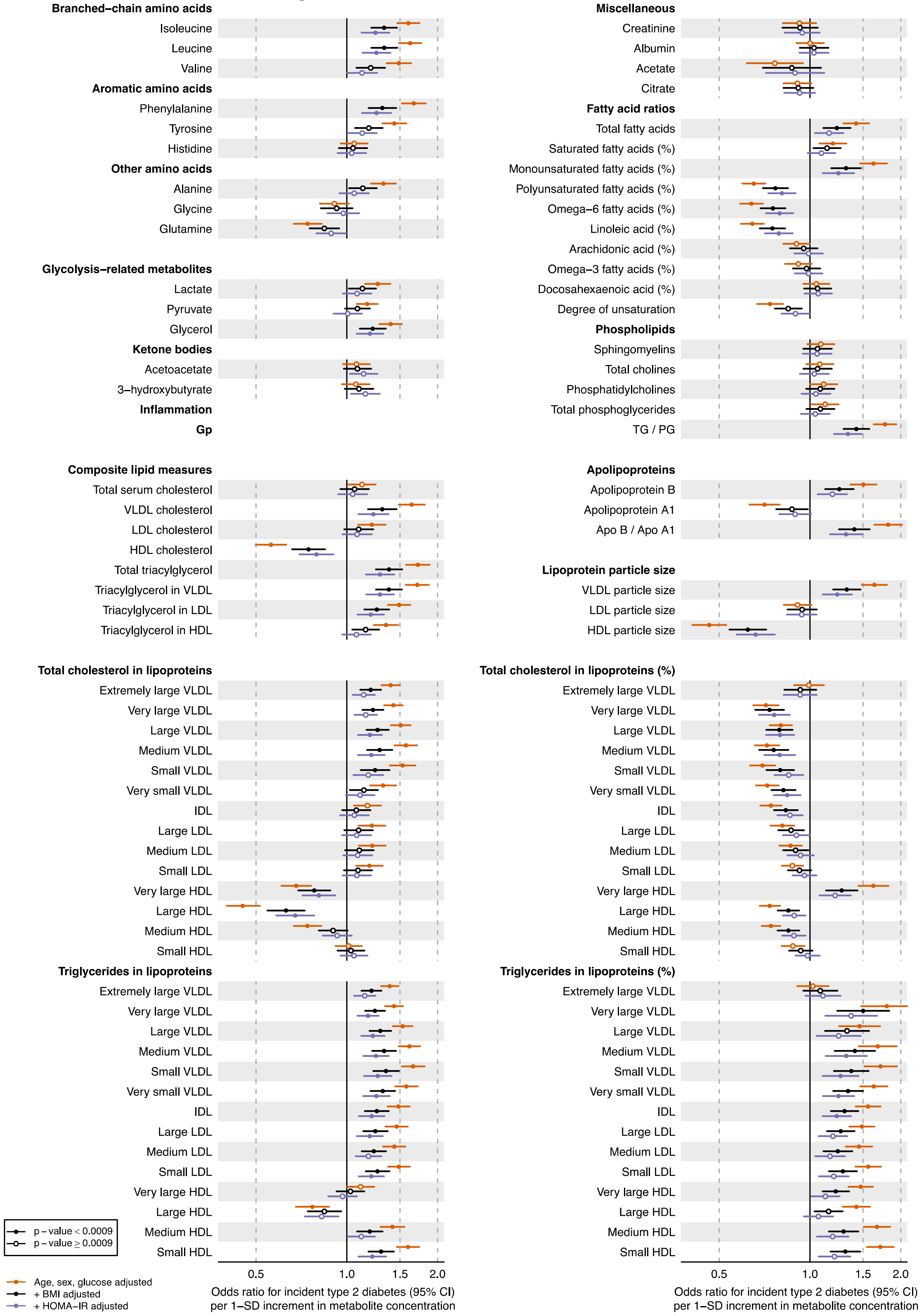

Values are odds ratios (95% confidence intervals) per 1-SD $\log_e$-transformed metabolite concentration. Odds ratios are shown without adjustment for baseline BMI and with adjustment for both BMI and HOMA-IR. All models were adjusted for sex, baseline age, and fasting glucose. The results were meta-analyzed for 11,896 young adults from the four cohorts.

ESM Fig 6. Correlations of overall biomarker association patterns for fasting glucose, 2h glucose and HOMA-IR at follow-up.

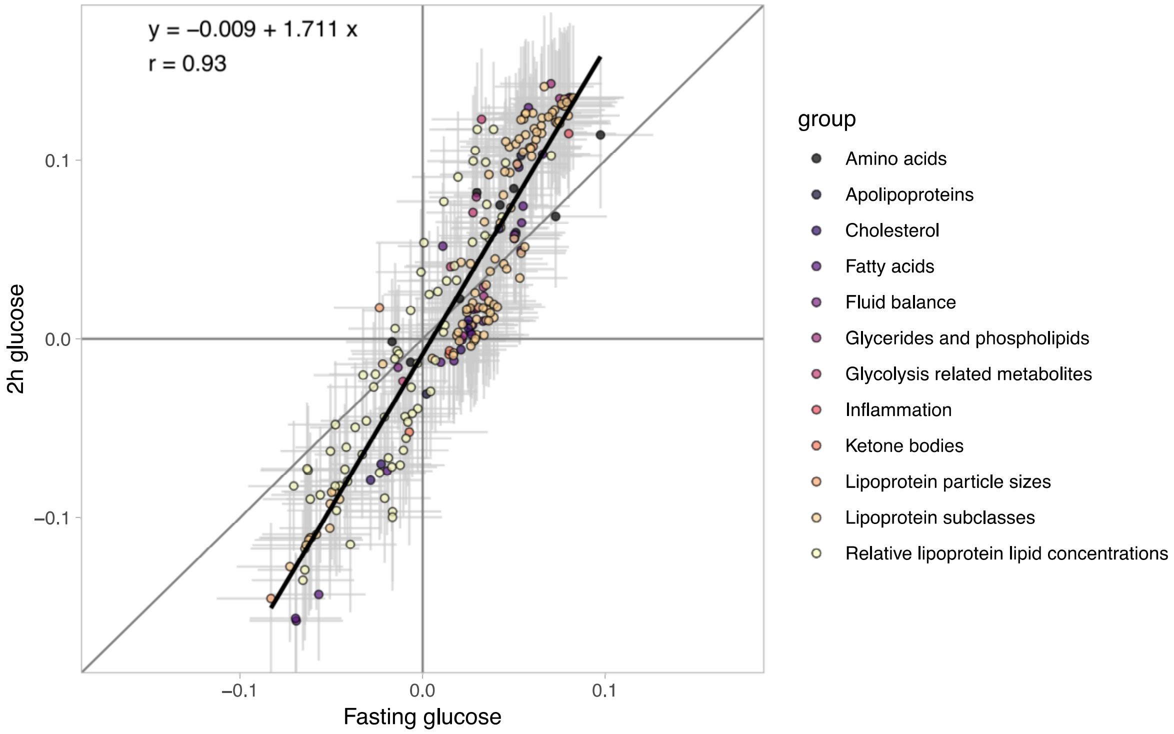

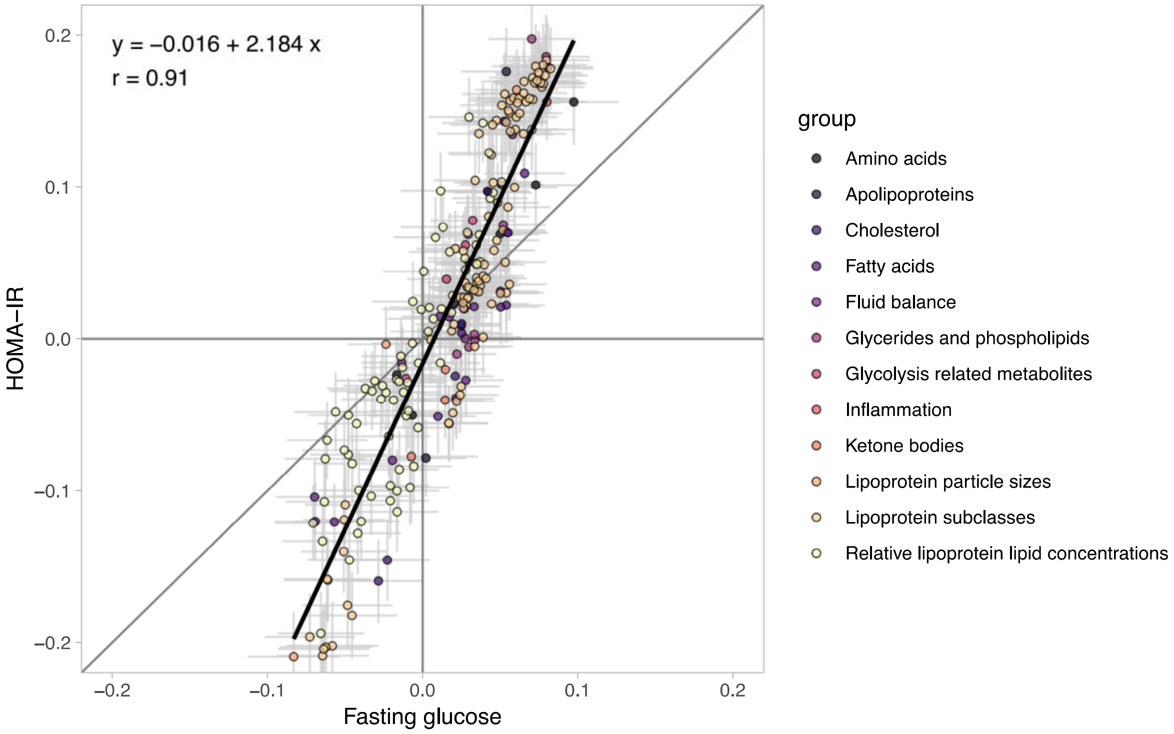

Comparison of metabolite associations for three different glycemic measures using linear comparison of the metabolic signature comprised of all 229 metabolic measures (introduced in Würtz et al, PLOS Med 2014;11:e1001765). The think black line indicates the linear fit between the metabolite associations comparisons. The slope indicates how much stronger (on average) are the associations with 2h glucose and HOMA-IR as compared to associations with fasting glucose. The correlation *r* signifies the overall similar of the patterns of metabolite associations.

ESM Fig. 7. Biomarker associations with incident impaired fasting glucose compared with incident type 2 diabetes.

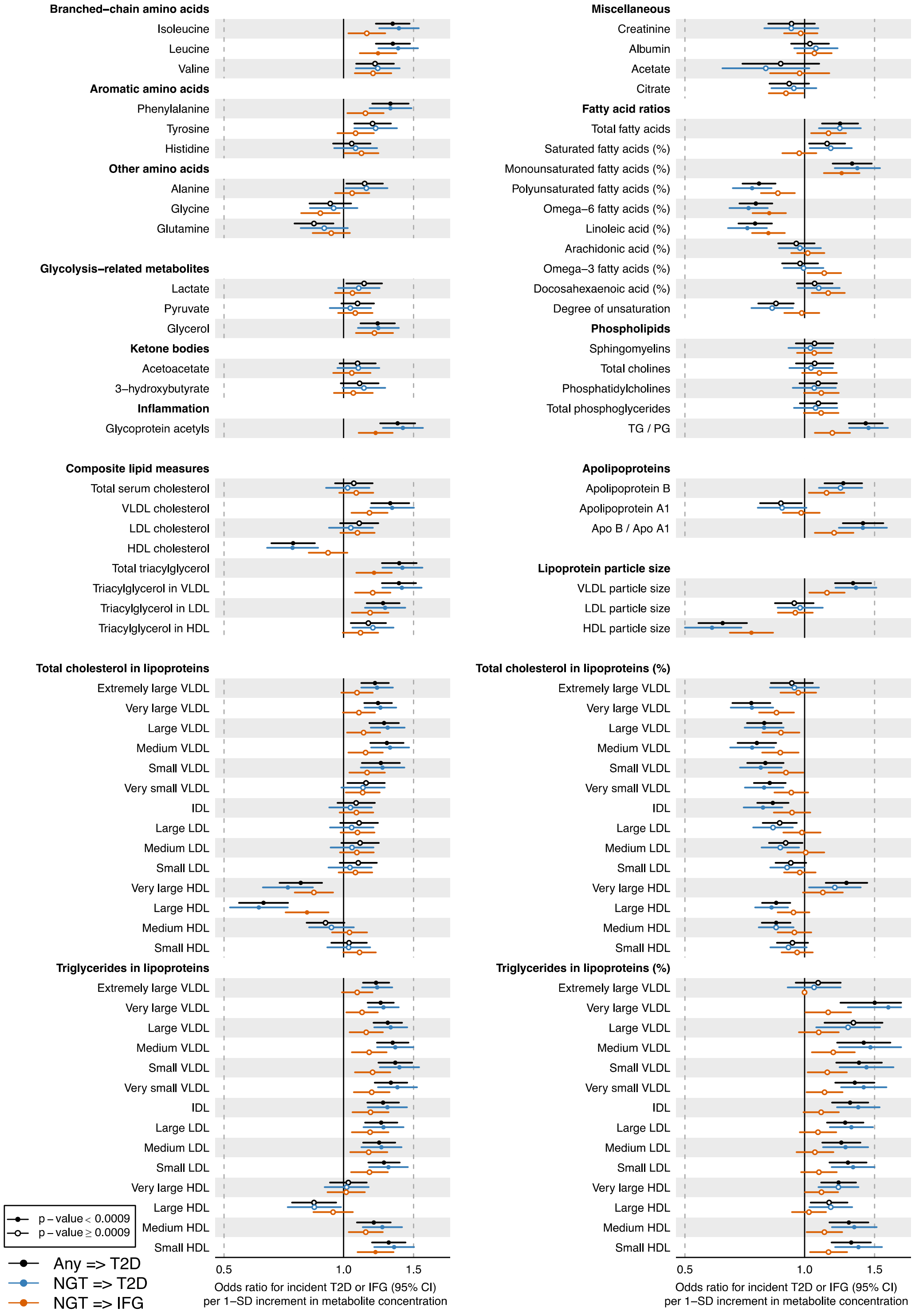

Values are odds ratios (95% confidence intervals) per 1 SD $\log_e$-transformed metabolite concentration shown a) incident type 2 diabetes in the whole study population (n=11,896; black), *b*) incident type 2 diabetes in the subset of individuals who had normal fasting glucose at baseline (n=8,729 with available data; 282 cases at follow-up; blue), and *c*) incident impaired fasting glucose in the subset of individuals who had normal fasting glucose at baseline (n=4,691 with available

data; 635 cases at follow-up; orange). All odds ratios were adjusted for baseline age, BMI, and fasting glucose. Results were meta-analyzed across the 3 cohorts with fasting blood samples.

ESM Fig. 8. Biomarkers for the risk of future diabetes compared to cross-sectional associations with BMI, HOMA-IR and fasting glucose.

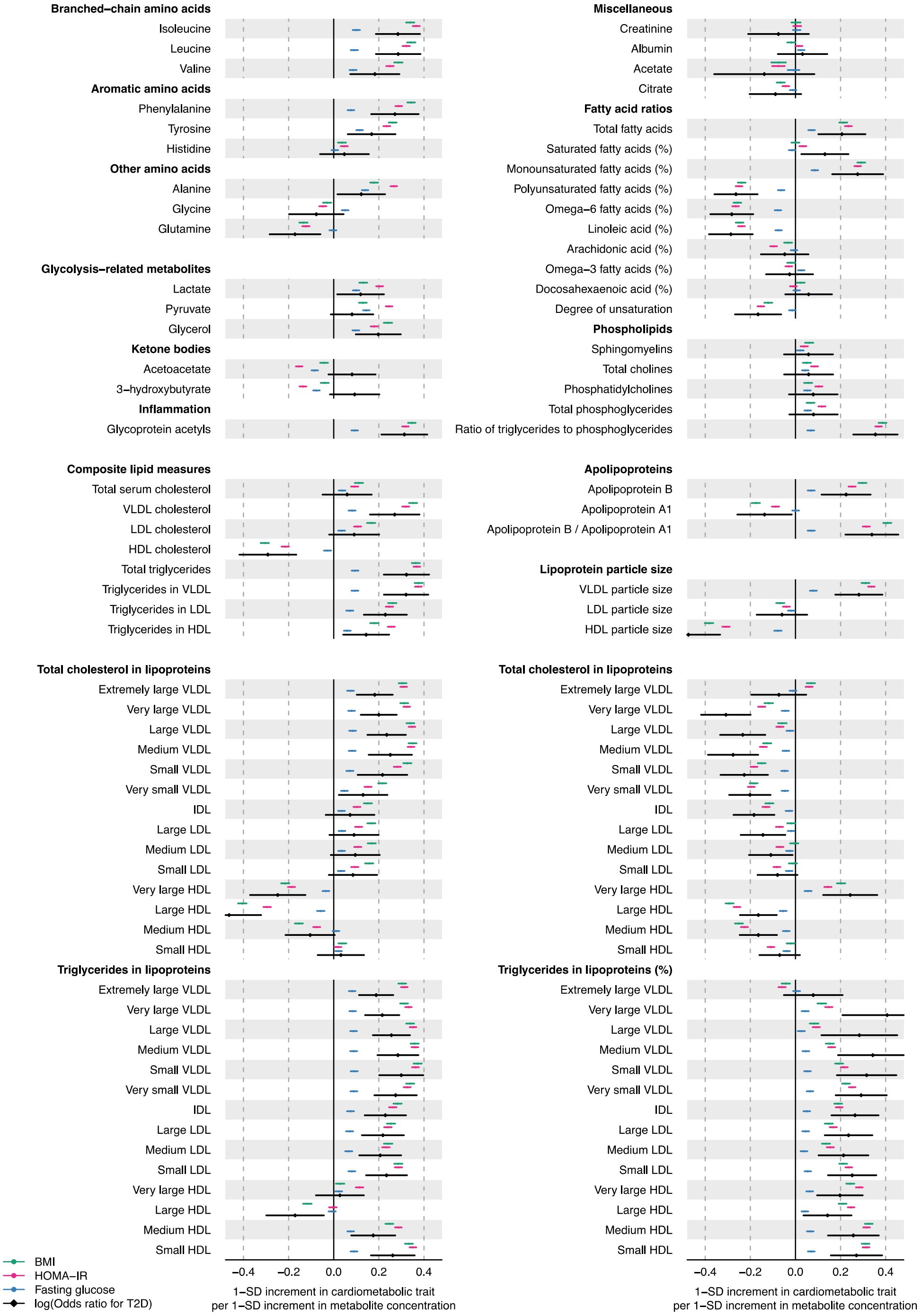

Values are beta coefficients from cross-sectional metabolite associations with BMI (green), $\log_e$(HOMA-IR) (red) and fasting glucose (blue). To enable comparison of the patterns of associations, magnitudes are scaled to 1-SD in each of the outcomes (corresponding to 4.2 kg/m^2 for BMI, 0.57 for $\log_e$(HOMA-IR) and 0.56 mmol/l for glucose) per 1 SD $\log_e$-transformed metabolite concentration. Also shown for comparison are the beta-coefficients of the main results for risk of future type 2 diabetes risk (=natural logarithm of odds ratio; black). Results were adjusted for sex and age, and meta-analyzed for 11,896 individuals from the four cohorts. Error bars

denote 95% confidence intervals; the large sample size and consistency across cohorts make confidence intervals narrow for the cross-sectional linear regression analyses.

ESM Fig. 9. Refinement of absolute risk for type 2 diabetes by including multi-metabolite score into prediction model.

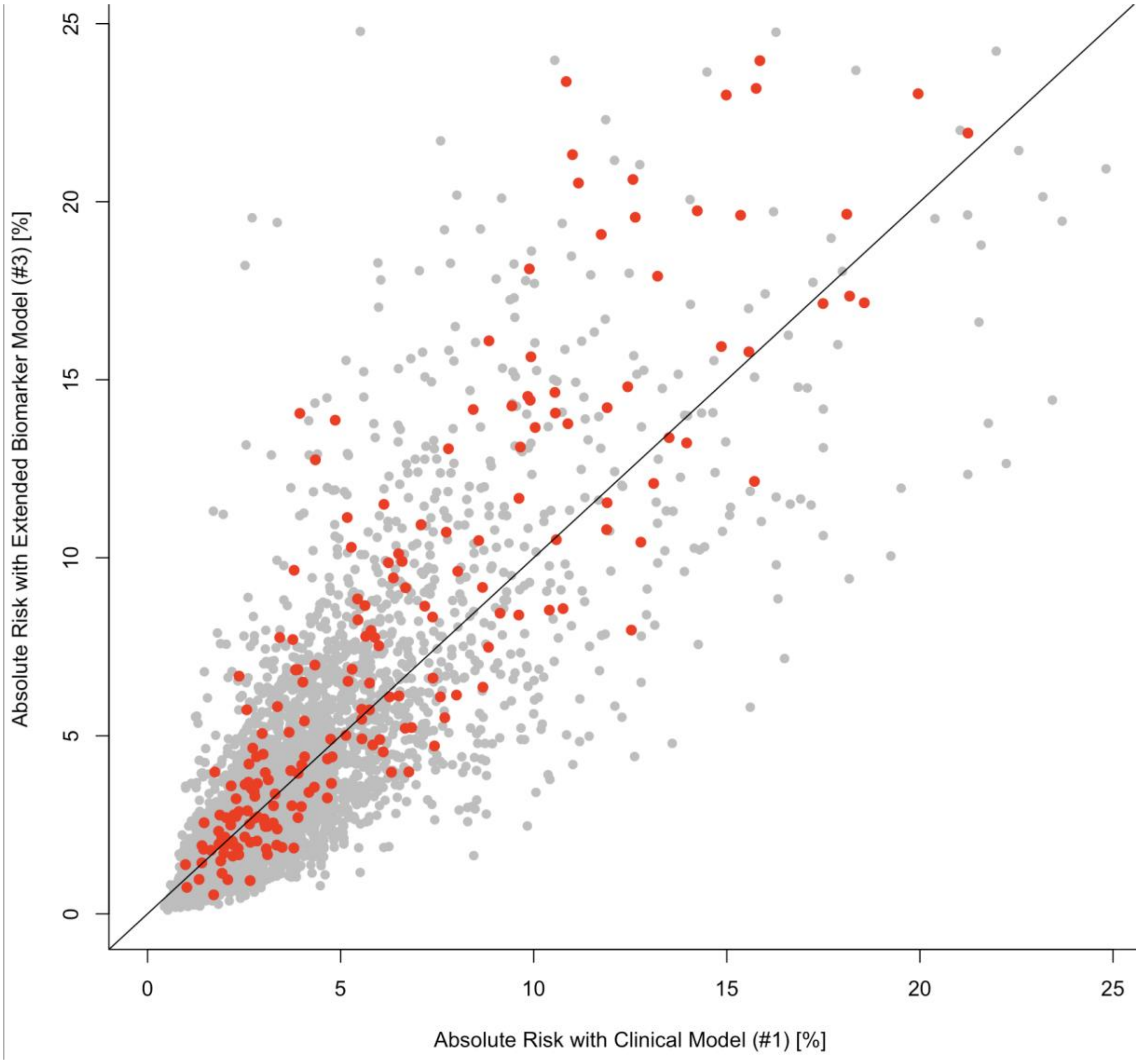

Illustration of the prediction of absolute risk for type 2 diabetes using the 'basic clinical model' compared to the 'biomarker-enhanced model'. The results were assessed in the NFBC validation cohort of 5,271 individuals, who were all 31 years of age at blood sampling. Red dots indicate individuals who developed type 2 diabetes during the 15-year follow-up period, and grey dots indicate those who did not develop type 2 diabetes.
